# Quantum interference enhances the performance of single-molecule transistors

**DOI:** 10.1038/s41565-024-01633-1

**Published:** 2024-03-25

**Authors:** Zhixin Chen, Iain M. Grace, Steffen L. Woltering, Lina Chen, Alex Gee, Jonathan Baugh, G. Andrew D. Briggs, Lapo Bogani, Jan A. Mol, Colin J. Lambert, Harry L. Anderson, James O. Thomas

**Affiliations:** 1 Department of Materials, University of Oxford, Oxford, UK; 2 Department of Physics, Lancaster University, Lancaster, UK; 3 Department of Chemistry, University of Oxford, Chemistry Research Laboratory, Oxford, UK; 4 Institute for Quantum Computing, University of Waterloo, Waterloo, Ontario Canada; 5 Departments of Chemistry and Physics, University of Florence, Sesto Fiorentino, Italy; 6 School of Physical and Chemical Sciences, Queen Mary University of London, London, UK

**Keywords:** Electronic devices, Electronic and spintronic devices, Materials for devices, Electronic properties and devices

## Abstract

Quantum effects in nanoscale electronic devices promise to lead to new types of functionality not achievable using classical electronic components. However, quantum behaviour also presents an unresolved challenge facing electronics at the few-nanometre scale: resistive channels start leaking owing to quantum tunnelling. This affects the performance of nanoscale transistors, with direct source–drain tunnelling degrading switching ratios and subthreshold swings, and ultimately limiting operating frequency due to increased static power dissipation. The usual strategy to mitigate quantum effects has been to increase device complexity, but theory shows that if quantum effects can be exploited in molecular-scale electronics, this could provide a route to lower energy consumption and boost device performance. Here we demonstrate these effects experimentally, showing how the performance of molecular transistors is improved when the resistive channel contains two destructively interfering waves. We use a zinc-porphyrin coupled to graphene electrodes in a three-terminal transistor to demonstrate a >10^4^ conductance-switching ratio, a subthreshold swing at the thermionic limit, a >7 kHz operating frequency and stability over >10^5^ cycles. We fully map the anti-resonance interference features in conductance, reproduce the behaviour by density functional theory calculations and trace back the high performance to the coupling between molecular orbitals and graphene edge states. These results demonstrate how the quantum nature of electron transmission at the nanoscale can enhance, rather than degrade, device performance, and highlight directions for future development of miniaturized electronics.

## Abstract (web summary)

An experimental demonstration of how destructive quantum interference effects can increase the performance of single-molecule field-effect transistors to reach levels similar to those of nanoelectronic transistors.

## Main

**Fig. 1:**
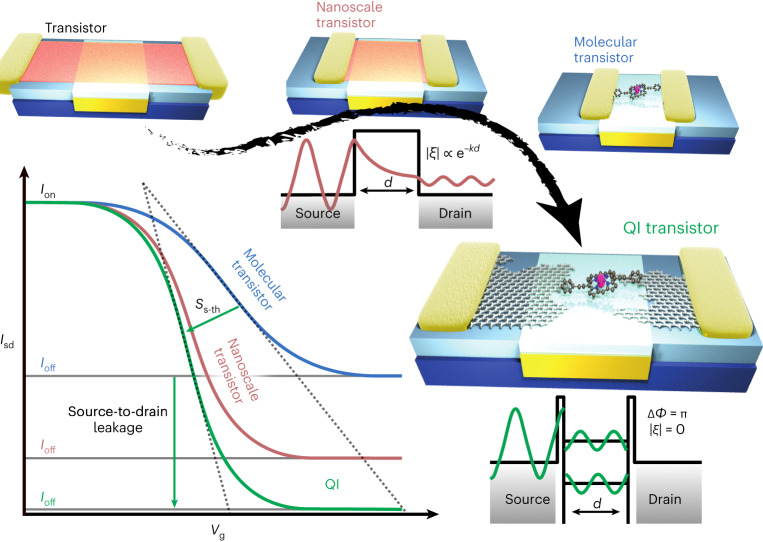
A QI-enhanced SMT. As the source-to-drain distance, *d*, of a transistor approaches the nanometre scale, quantum-tunnelling-mediated transmission (*ζ*) through the potential energy barrier that creates an off state increases exponentially, leading to high leakage current and degrading the device subthreshold swing (*S*_s-th_). The source–drain leakage becomes increasingly problematic at the molecular scale (<5 nm) unless interference between two coherent conduction channels acts to suppress transmission. For two quantum-coherent transport channels (with transmission coefficients *ζ*_1_, *ζ*_2_, where $\zeta_i=|\zeta_i|{\mathrm {e}}^{-i{{\phi}_i}}$), total transmission can be completely suppressed if |*ζ*_1_| = |*ζ*_2_| and their phase difference, Δ*ϕ* = π (through *ζ*^2^ = |*ζ*_1_ + *ζ*_2_|^2^ = |*ζ*_1_|^2^ + |*ζ*_2_|^2^ + 2|*ζ*_1_||*ζ*_2_|cos Δ*ϕ*), providing a route to regain desirable characteristics of mesoscopic transistor geometries even with a few-nanometre channel length.

Tunnelling field-effect transistors^1^ and single-molecule transistors (SMTs)^2^ are devices where quantum effects in electron transmission, normally considered to be detrimental to the performance of transistors with nanometre dimensions^3^, become responsible for the function of the device. Using a single molecule as an active channel brings the benefit of synthesis with atomic precision, providing the possibility to control quantum effects through molecular design to enable high performance^4^, as well as leading to complementary functionalities such as thermoelectric recovery of waste heat, multistate switching or sensing^5^. Quantum interference (QI) is a characteristic quantum effect found in nanoscale charge transport and has been predicted to enhance transistor performance (Fig. 1)^6–13^. However, it is difficult to create two nanoscale quantum-coherent channels in standard conductors, because scattering and defects quickly lead to loss of electron coherence. Consequently, the practical use of QI has been almost exclusively limited to superconducting devices to obtain extremely sensitive magnetometers^14^, and its potential for transistors remains largely unexplored. Nevertheless, theory predicts that harnessing it is a promising route to high-performance SMTs or efficient thermoelectric generators^6^. Evaluating these predictions requires fundamental experimental investigation into the specific impact of QI on transistor properties.

Destructive QI (DQI) can be controlled by electrochemical gating, whereby conductance switching over two orders of magnitude has been achieved for several cycles^9,10^. However, electrochemical gating is relatively slow and incompatible with many practical applications. Graphene source and drain electrodes enable more versatile electrostatic gating and measurement of QI in single-molecule devices^15^. Furthermore, non-trivial transmission effects resulting from the coupling between the graphene density of states with molecular orbitals can sometimes enhance the device properties^16,17^.

**Fig. 2:**
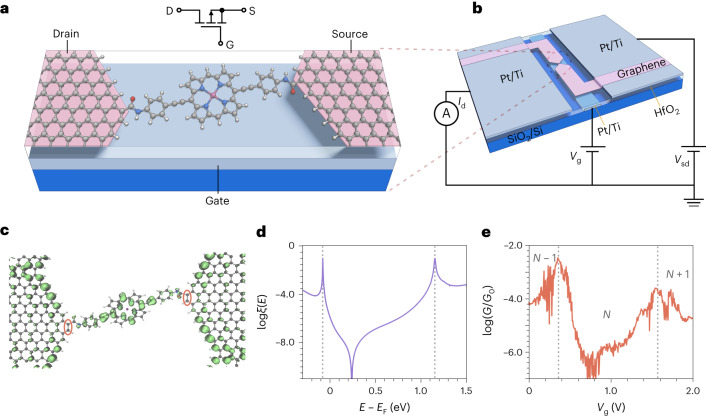
Transistor architecture and QI-mediated transmission. **a**, Schematic representation of a graphene-based SMT. The 3,5-bis(trihexylsilyl)phenyl solubilizing groups on the lateral *meso* positions of the porphyrin have been replaced with H atoms for clarity. **b**, Device architecture. The grey-blue rectangular strip in the centre is the local platinum gate electrode under a 10 nm layer of HfO_2_ (transparent); the rectangular areas (grey-blue) at each end are the source and drain platinum electrodes, which are in contact with the bow-tie-shaped graphene (pink). **c**, Optimized junction geometry with the LDOS at the Fermi level shown in green (the isovalue is set at 0.0005). The zero-LDOS carbon atoms are highlighted in red. **d**, Calculated behaviour of the electronic transmission *ξ* as a function of energy. **e**, Differential conductance at *T* = 80 K versus *V*_g_ at *V*_sd_ = 0 mV. The conductance is plotted on a logarithmic scale as the ratio to conductance quantum, *G*_0_.

To explore the use of QI in nanoelectronic devices, we employ a zinc-porphyrin with 4-ethynylaniline anchor groups at opposite (5,15) *meso* positions and bulky 3,5-bis(trihexylsilyl)phenyl substituents at the other two (10, 20) *meso* positions as an active channel (Fig. 2a; see Supplementary Section 1 for synthesis). The energy-level spacings and the chemical potentials are found to be within the experimental ranges of source–drain and gate voltages (*V*_sd_ and *V*_g_, respectively). As a result, both the on- and off-resonance transport regimes are accessible, allowing evaluation of not only interference between orbitals but also the influence of their coupling to the reservoirs. The molecules were integrated into three-terminal molecular transistor devices by direct covalent coupling of amine groups to carboxylic acid residues on the oxidized edges of graphene electrodes, which are generated during electroburning (Methods)^18^. The current *I*_sd_ is measured on applying *V*_sd_, and the device behaviour can be switched using *V*_g_ (Fig. 2b).

## Quantum interference

The atomically defined nature of the molecular transport channel allows the prediction of the device behaviour using a combination of density functional theory (DFT) and quantum transport theory^19,20^. For the isolated molecule, the highest occupied molecular orbital (HOMO) and the lowest unoccupied molecular orbital (LUMO) are not predicted to interfere destructively through symmetry^21^ or connectivity^22^ arguments (Supplementary Section 8). Instead, electronic interference at the graphene edges leads to DQI effects, causing zeroes in the local density of states (LDOS) at the Fermi energy at irregular edges, as shown by the red circles in Fig. 2c and in refs. ^16,23^. Molecular coupling between the edges of the graphene source and drain not only provides orbital resonances where device transmission is high but also maps these energy-dependent DQI effects onto the overall transmission spectrum (Fig. 2c,d) to give regions of suppressed conductance, shown in spatial visualization of the transmission pathway^24^ (Supplementary Section 8). The resulting full energy-dependent (*E*) electron transmission spectrum, *ξ*(*E*), spans over ten decades, with an asymmetric shape and an extremely pronounced dip produced by anti-resonance within the HOMO–LUMO gap (Fig. 2d). This is a surprising result: contrary to the usual case where QI is produced by the phase properties of the frontier molecular orbitals^21^, the anti-resonance here arises as a result of coupling between graphene edge states via molecular orbitals (Supplementary Section 8). Since the vanishing of the LDOS is a generic feature of the lead, the DQI dip should remain robust to changes in the orientation of the molecule and electrode–electrode distance (provided that through-space tunnelling is negligible).

The experimental behaviour shows the predicted features, as shown by plotting the gate dependence of the zero-bias conductance (normalized by the conductance quantum)^11^, *G*_sd_ = *∂I*_sd_/*∂V*_sd_ ∝ *ξ*(*E*) (Fig. 2e) of a device (device 1; see Methods and Supplementary Table 3-1 for data on other devices). The peaks in the conductance trace arise from resonant charging of the molecule, and at *V*_g_ ≈ 0.4 V, transport occurs via a change in the occupation of the HOMO, while at *V*_g_ ≈ 1.6 V, the LUMO dominates transport, as detailed later in the full single-electron transistor characterization. The asymmetric anti-resonance feature predicted by the calculations is observed in the region between these peaks where the molecule remains neutral, showing a pronounced dip arising from the DQI effect. For a large region around *V*_g_ = 0.78 V, the conductance at the dip reaches down to below the lowest detection level of our set-up, ~10^−7.0^ *G*_0_, where *G*_0_ is the conductance quantum. This demonstrates that modulating *V*_g_ moves the molecular levels of the porphyrin between being on-resonance, where conductance is at local maxima, to off-resonance and complete conductance suppression.

## Unimolecular transistor characteristics

**Fig. 3:**
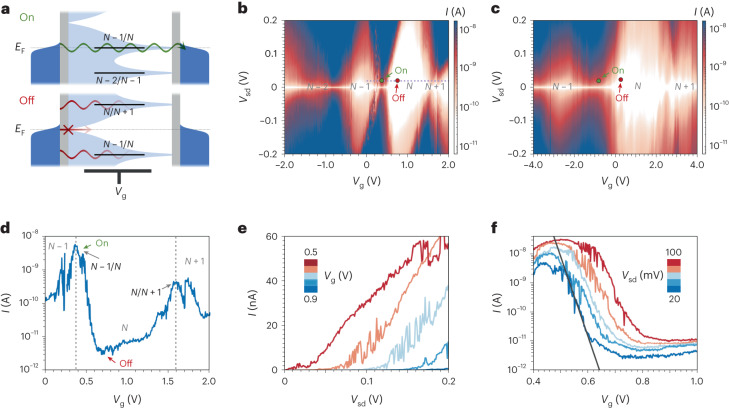
QI transistor properties. **a**, Schematic model of the QI-based transistor, where conductance is high when a molecular level is on resonance, and low when a phase difference between two pathways suppresses transmission in the *N* state (the HOMO–LUMO gap). **b**,**c**, Current map (*I*_sd_ versus *V*_sd_ and *V*_g_) for devices 1 (**b**) and 2 (**c**). Device 1 is discussed in detail in the main text; devices 2–4 are characterized in Supplementary Fig. 3-4. **d**, *I*_sd_–*V*_g_ at *V*_sd_ = 20 mV (along the dashed line in **b**). **e**,**f**, Output characteristics (**e**) and transfer characteristics (**f**) of the single-porphyrin transistor; the slope of the grey line shows the thermionic limit at the measurement temperature. All measurements were done at 80 K.

The QI effects can now be used to operate a transistor device. When charging is considered, the full behaviour includes neutral molecular states, with *N* electrons, and oxidized (*N* – 1) and reduced (*N* + 1) states produced by varying *V*_g_. High on/off current ratios are afforded by using the DQI-induced conductance dip as the off state and the *N* – 1/*N* resonant tunnelling channel as the on state (Fig. 3a). The full mapping of the current versus *V*_sd_ and *V*_g_ shows reproducible single-electron-transistor behaviour (Fig. 3b,c) and locally steep responses of current to gate voltage, which are advantageous for the efficient switching of the device (Fig. 3d, device 1, and Supplementary Section 3 for additional devices). Regions of low current are when the device is in the off-resonant condition where first-order tunnelling processes are suppressed by Coulomb blockade, and low residual current is carried by phase-coherent off-resonant transmission. These regions (known as Coulomb diamonds) are separated by the resonant tunnelling regions, when a molecular level is within the bias window generated by applying *V*_sd_, where current is high (Fig. 3b). The number of electrons on the molecule is fixed within each Coulomb diamond and varies by one between adjacent diamonds^2^, leading to the definitions of the *N* – 1, *N* and *N* + 1 states mentioned above. The *N* – 1/*N* transition occurs at positive gate voltage (*V*_g_ = 0.4 V) and varies slightly between devices, indicating that there is the possibility of charge transfer from the molecule to the graphene at zero gate voltage. This effect has been observed previously for single-molecule devices^25^ and is because of p-doping of the graphene by the underlying substrate^26^, bringing the Fermi level close to, or below, the first oxidation potential of the porphyrin. The coexistence of Coulomb blockade and phase-coherent off-resonant tunnelling demonstrates that the device is in the intermediate molecule–electrode coupling (*Γ*) regime^27,28^, and from the full width at half maximum of a Coulomb peak, we find *Γ* = 8 meV.

In Fig. 3e,f, we focus on the transistor performance of the device through plots of the output (*I*_sd_–*V*_sd_) and transfer characteristics (*I*_sd_–*V*_g_) between the on and off states. Within the dip (*V*_g_ = 0.9 V), Coulomb blockade and DQI lead to extremely low currents with a resistance of ~1 GΩ, whereas on the *N* – 1/*N* resonance (*V*_g_ = 0.5 V), resonant tunnelling via the porphyrin HOMO leads to an *I*_sd_–*V*_sd_ that is approximately linear with a resistance of ~3 MΩ (Fig. 3e). Shifting the gate potential to move the device between on and off states leads to a current ratio of 10^3^–10^4^, depending on *V*_sd_, and despite the differences in switching mechanism between typical MOSFETs and our SMT, the transfer characteristics have a similar shape (Fig. 3f). There is an approximately linear increase in log_10_(*I*_sd_) as *V*_g_ is swept from off to on before a saturation of the source–drain current; from these data, we calculate the subthreshold swing (*S*_s-th_) of the single-molecule device. We find a value of *S*_s-th_ = 14.5 ± 0.4 mV dec^−1^ at *V*_sd_ = 20 mV and 80 K after adjusting for the unoptimized gate coupling parameter, *α*_g_. The value is very close to the thermionic limit—the lower limit on the subthreshold swing for a MOSFET (15.9 mV dec^−1^ at 80 K) that we attribute to the steepness of the transmission conferred by DQI.

## Temperature-dependent behaviour

**Fig. 4:**
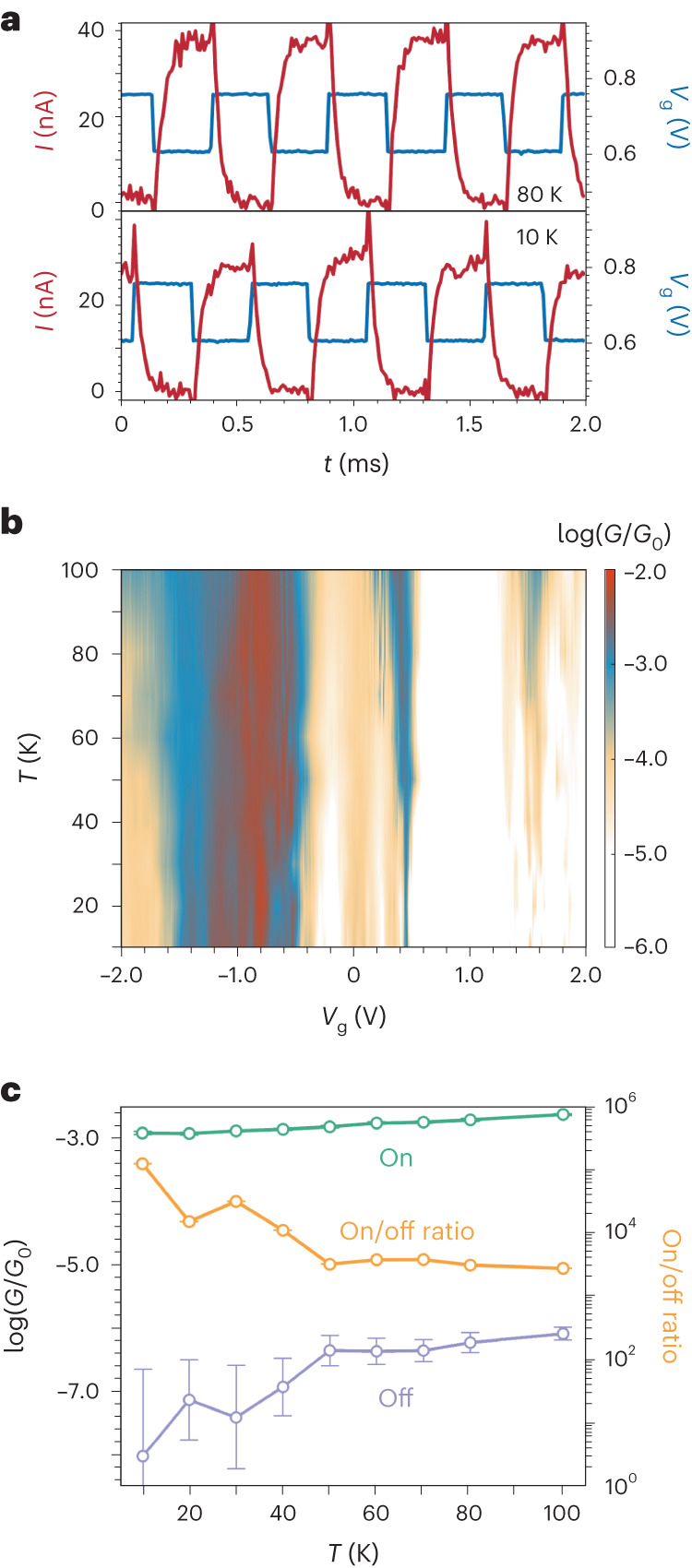
Switching and temperature-dependent behaviour at the thermionic limit. **a**, On/off switching of the device with a 2 kHz square wave applied to the gate at *V*_sd_ = 100 mV at 80 K (top) and 10 K (bottom). Longer time traces and intermediate temperatures are shown in Supplementary Fig. 6-1. **b**, Differential conductance map measured as a function of temperature and gate voltage at *V*_sd_ = 0 mV. **c**, Conductance for the on state (green), the off state (purple), and the conductance on/off ratio (orange) at *V*_sd_ = 20 mV. The error bars represent the standard deviation of the conductance in a 50 mV gate-voltage window (19 data points) around the on- and off-state value.

We test the switching frequency limits of the device by applying a square wave to the gate (*V*_g,min_ = 0.61 V, *V*_g,max_ = 0.76 V) with a fixed bias voltage of *V*_sd_ = 100 mV. Robust current switching is observed at kilohertz frequencies (Fig. 4a and Supplementary Fig. 4-1). The response of the current to the voltage is rounded by the RC (resistance × capacitance) time, *τ*, of the circuit. In this case, *τ* = 30 ± 1 μs, giving a rise/fall time (10% to 90% of on-state current for rise time, vice versa for fall time) of 2.2 × *τ*_rise/fall_ = 66 ± 2 μs and a maximum switching frequency (10% to 90%) of 1/(*τ*_rise_ + *τ*_fall_) = 7.6 ± 0.3 kHz. The frequency is limited by the bandwidth of the current amplifier, rather than the intrinsic switching mechanism of the molecular device, as the same RC time is found by measuring the output of the circuit with a 100 MΩ resistor in place of the molecular device (Supplementary Fig. 4-1). As shown by the agreement between the transmission spectrum calculated by non-interacting Landauer theory (in which the molecular geometry is fixed) and the experimental zero-bias conductance (Fig. 2d,e), the switching mechanism is a transition from DQI-suppressed off-resonant transmission to resonant tunnelling with gate voltage and does not involve any specific conformational changes of the molecule. As sequential resonant tunnelling is first order in *Γ*, we estimate that the intrinsic switching frequency could be up to *hf* ≈ *Γ* ≈ 8 meV (found from fitting the left side of the resonance peak) around 1 THz. Therefore, the upper frequency limit of switching for the actual device will be limited by the time taken to charge the gate, a property that depends on the dimensions and material of the electrode. Optimizing the gate specifications is required for future device development but beyond the scope of our study of the effect of DQI on molecular transistor performance.

A map of *G*_sd_ versus *T* shows that the on-state resonance width remains constant below 30 K and then broadens on increasing *T* because of temperature effects on the electrode Fermi distributions (Fig. 4b). We also observe that the conductance on resonance begins to increase above 30 K, suggesting that thermally activated processes begin to contribute to the conduction mechanism (Fig. 4c and Supplementary Fig. 7-1). We limit our analysis to temperatures in the range of 10–100 K because of the difficulty of accounting for charge trapping and atomic-scale fluctuations at graphene edges at higher temperatures^29,30^. Progress in controlling these effects is crucial for increasing the operating temperature^31^. The DQI dip, measured at *V*_g_ ≈ 0.8 V, where the transport mechanism is solely off-resonance phase-coherent transport, remains close to our minimum detection limit in the *T* = 10–100 K region. The slight positive *T* dependence is probably the result of a reduced influence of the DQI-induced suppression, which relies on coherent electron transmission, owing to dephasing produced at higher temperatures by inelastic tunnelling and vibrations at the dynamic molecular/graphene interfaces^32^. The positive *T* dependence of both on and off states leads to a stable on/off ratio above 40 K.

## Transistor performance and limits

**Fig. 5:**
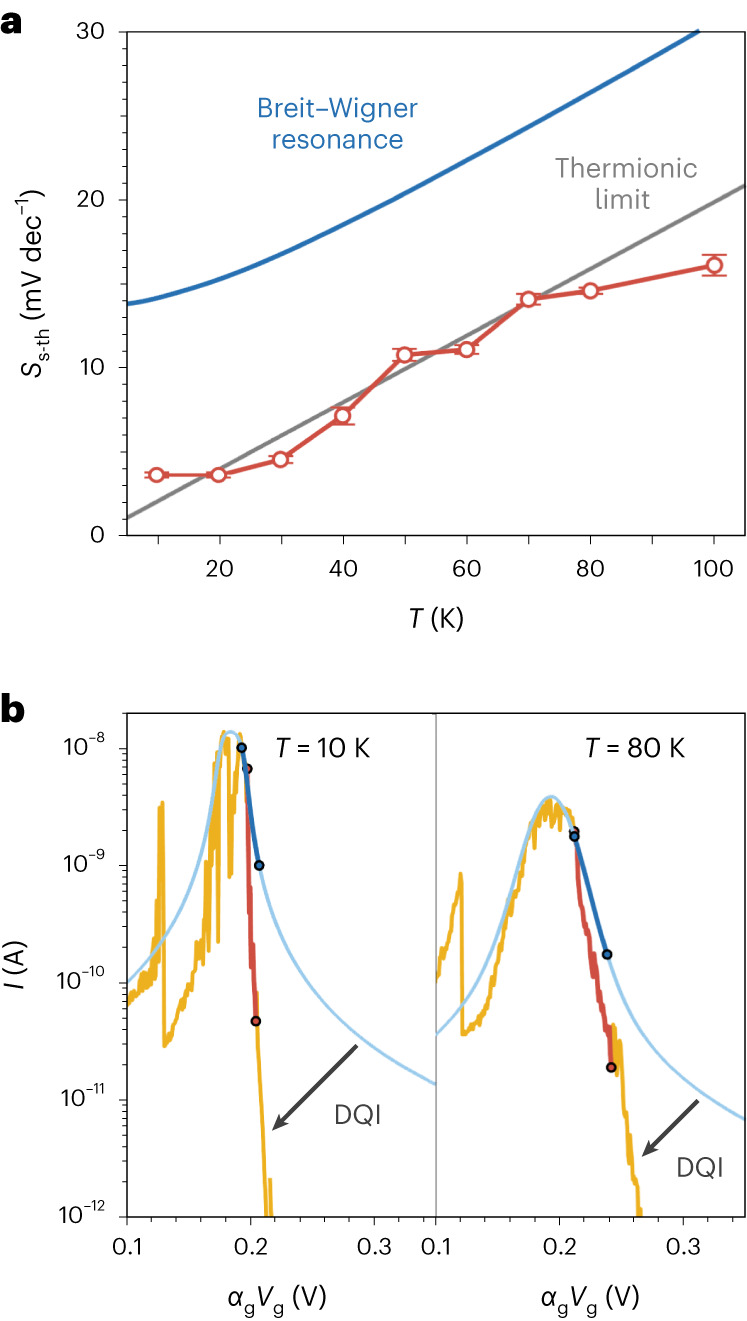
Subthreshold swing of a QI-enhanced transistor. **a**, Normalized subthreshold swing at *V*_sd_ = 20 mV as a function of temperature (red circles; see Supplementary Figs. 5-1–5-9 for *S*_s-th_ calculation), plotted with the thermionic limit for a classical field-effect transistor (grey), and simulated subthreshold swing for a single-level molecular Breit–Wigner resonance without DQI (blue). **b**, *I*_sd_ as a function of *α*_g_*V*_g_ around the *N* *–* 1/*N* resonance; the yellow curves are experimental and the blue curves are the simulations of a Breit–Wigner resonance. The ranges to calculate the subthreshold swing are the steepest parts of the curves and are highlighted (red for experimental and blue for simulated) and demonstrate the effect of DQI to increase the magnitude of the gradient of the *N* state current, thereby reducing *S*_s-th_ to the thermionic limit.

We now examine, through the subthreshold swing, how efficient switching of the device can be achieved. The best gate control that can be achieved in a field-effect transistor is the thermionic limit that results from the exponential tails of the Fermi distributions of the electrodes (Fig. 5a)^33^; however, in nanodevices, performance is usually degraded further by short-channel parasitic effects. The tunnelling current in a single-molecule junction is the convolution of *ξ*(*E*) with the difference in electrode Fermi distributions ${I}_{\mathrm{sd}}\propto \int \xi (E)[\,{f}_{\mathrm{S}}({\mu }_{\mathrm{S}})-{f}_{\mathrm{d}}({\mu }_{\mathrm{d}})]{{\mathrm{d}}{{E}}}$, and so a limit on *S*_s-th_ related to *T* will also apply. To understand the effect of DQI on this limit, we compare our experimental measurements of *S*_s-th_ at different temperatures to a simulation of *S*_s-th_ versus *T* around *N* – 1/*N* using the single-level model^27^. The single-level model treats the molecular resonance as a Breit–Wigner resonance, and as it only considers transmission through a single channel, it cannot capture interference effects. The lifetime broadening conferred by *Γ* (= 8 meV, the experimentally derived value) and thermal broadening both contribute to the subthreshold swing in an SMT, ensuring that it remains above the thermionic limit at all *T*, with a small change where *k*_B_*T* ≪ *Γ* (where *k*_B_ is the Boltzmann constant) that becomes linear as *T* increases (Fig. 5a). This result reveals a fundamental trade-off when designing a three-terminal nanodevice for transistor applications: a larger *Γ* is desirable to give high on-state currents but comes at the expense of higher off-state currents and larger subthreshold swings. Conversely, a smaller *Γ* puts *S*_s-th_ closer to the thermionic limit but reduces tunnelling currents to smaller values, leading to high resistances on resonance, thereby limiting on/off ratios. Utilizing DQI removes the need to compromise: DQI suppresses off-resonant phase-coherent transport, leading to the steep energy-dependent transmission through the molecular device, even with an intermediate *Γ*, thereby permitting a high on-state and low off-state current that can be switched by only a small change in *V*_g_.

This effect is demonstrated here explicitly in Fig. 5b, where DQI accounts for the difference between the simulated and experimental data. At 10 K, DQI reduces *S*_s-th_ from 14.1 mV dec^−1^ to 3.6 ± 0.4 mV dec^−1^ (thermionic limit 2.0 mV dec^−1^), and at 80 K, from 26.3 mV dec^−1^ to 14.5 ± 0.4 mV dec^−1^ (thermionic limit 15.9 mV dec^−1^). These observations show the value of DQI in the device performance: it effectively negates the additional contribution of lifetime broadening to the subthreshold swing, reducing the value to the thermionic limit, even in the intermediate coupling regime.

**Extended Data Fig. 1:**
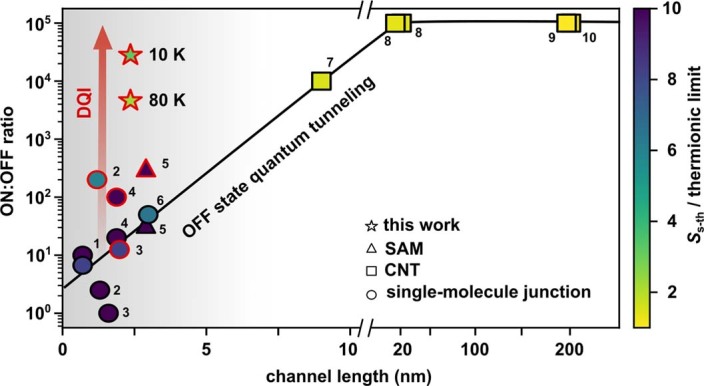
Overview of quantum-Interference-enhanced transistor performance. Scatter plot of ON/OFF ratio and molecular lengths for a number of molecular and carbon nanotube transistor devices (**1**^48^, **2**^9^, **3**^49^, **4**^10^, **5**^50^, **6**^51^, **7**^52^, **8**^53^, **9**^54^, **10**^55^ and this work, with the normalization for α_g_ removed); SAM = self-assembled monolayer, CNT = carbon nanotube. Marker colours represent the device subthreshold swing normalised by the thermionic limit at the measurement temperature, which is room temperature except for those specifically marked. Markers with red outlines represent devices that have DQI in their transmission, the black line below 10 nm is a fit to the data points without interference.

The general relationship between the on/off ratio and the channel length in sub-10 nm transistors (Extended Data Fig. 1) can be understood by considering the molecule as a quantum tunnelling barrier—even if the device is tuned to the off state (that is, modulating *V*_g_ to give a high tunnel barrier), devices inevitably become exponentially more transmissive with decreasing molecular length (that is, decreasing barrier width), raising off currents and decreasing the switching ratio. Without considering the phase-coherent nature of electron transmission, this leakage current fundamentally limits the transistor performance on this length scale, shown by the fit in Extended Data Fig. 1. If devices have DQI in their transmission functions, however, this need not be the case; low off currents, even with short (<1 nm) molecular lengths^34^, are possible if two out-of-phase coherent transport channels (such as edge states or molecular orbitals) can interfere to suppress overall device transmission. Extended Data Fig. 1 demonstrates this effect where devices at the scale of a few nanometres that utilize DQI can have properties of those with longer channel lengths. Utilizing this mechanism is a particular advantage of molecular nanoelectronics, as the energies and phase properties of orbitals are routinely engineered through molecular design and synthesis. The reduction in the on/off ratio with increasing temperature owing to increasing leakage current (Fig. 4c) is attributed to a partial quenching of interference effects^32^; however, a fuller understanding of the underlying mechanism is crucial to design devices that retain extremely low off currents resulting from DQI at room temperature. The numbers that we report compare favourably to previous studies of SMT properties that have yielded subthreshold swings in the range of 400–1,000 mV dec^−1^ (our value would be 140 mV dec^−1^ at room temperature, removing the adjustment for *α*_g_ and linearly extrapolating from Fig. 5a). While most SMTs are characterized by a handful of cycles at a frequency below a few hertz owing to inherent timescales of electrochemical gating, we demonstrate a switching frequency of ~7 kHz, and over the course of our measurements, we switch our device over 10^5^ times. Furthermore, previous measurements are mostly based on scanning tunneling microscopy break-junction techniques, so they involve continuous reforming of molecular junctions, with the conductance of on and off states taken as an average over many traces, rather than our measurements of repeatedly switching the same, static unimolecular device.

## Conclusion

Overall, these results reveal how QI can be harnessed in devices just a few nanometres wide, in pursuit of low-power miniaturized electronics. The performances attained offer proof of concept of nanodevices in which quantum effects are used as a resource to enhance device function, rather than being a limitation. Our demonstration makes specific use of the density of states fluctuations at graphene edges, revealing a hitherto undisclosed difference from standard metal electrodes. The key challenge that remains is to develop experimental methods to prepare graphene interfaces with defined geometries, such as through Joule heating^35^ or coupling to chemically synthesized nanoribbons^36,37^. The concepts we have presented, using phase-coherent transmission, can however be translated to a series of new compounds and device architectures that are designed to optimally exploit QI. The mild fabrication method allows using a wide range of chemical compounds to create these nanoscale transistors, opening the path to the creation of multifunctional devices, for example, with optical or spintronic properties, where interference can be used to control multiple effects at the same time.

## Methods

### Substrate fabrication

On an n-doped silicon wafer with a 300-nm-thick SiO_2_ layer, the gate electrode (3 μm wide) was defined by optical lithography with lift-off resist and electron-beam (e-beam) evaporation of titanium (5 nm) and platinum (15 nm). Next, an atomic-layer-deposition-grown dielectric layer of HfO_2_ (10 nm) was deposited. Finally, source and drain contact electrodes separated by a 7 μm gap (gap centred with the gate) were defined by optical lithography with lift-off resist and e-beam evaporation of titanium (5 nm) and platinum (45 nm). Wafers were diced into 10 × 10 mm chips, each containing 874 devices.

### Graphene patterning

Poly(methyl methacrylate) (PMMA)-protected chemical-vapour-deposition-grown graphene was wet-transferred to the substrate. The PMMA was removed in warm acetone for 3 h. The graphene tape with a bow-tie-shaped structure was patterned by e-beam lithography with bilayer lift-off resist (PMMA495 and PMMA950) and thermal evaporation of aluminium (50 nm). After lift-off, the graphene on unexposed areas (not covered by aluminium) was etched with oxygen plasma. The aluminium was subsequently removed by aqueous NaOH solution (1.0 g in 50 ml water). The sample was finally immersed in warm acetone overnight to remove any residual PMMA. Scanning electron microscopy images can be found in Supplementary Section 2.

### Graphene nanogaps

Graphene nanogaps were prepared by feedback-controlled electroburning of the graphene bow-tie shape until the resistance of the tunnel junction exceeds 1.3 GΩ (10^−7^ *G*_0_) (see Supplementary Section 2 for electroburning curves)^38,39^. HfO_2_ has a high dielectric constant, and in combination with the weaker screening of the gate field by using graphene in place of bulky three-dimensional metallic electrodes (most commonly used in molecular junctions)^40^, this yields a high gating efficiency. The empty nanogaps were characterized by measuring a current map as a function of bias voltage (*V*_sd_) and gate voltage (*V*_g_) at room temperature to exclude devices containing residual graphene quantum dots^41^. Only clean devices were selected for further measurement (see Supplementary Figs. 3-1, 3-3 and 3-6 for before and after current maps of the devices presented here).

### Molecular junctions, measurements and device numbers

The mechanism of graphene breakdown under electroburning in air is oxidation^18^, and the oxygen-containing functional groups that are thus formed at the edges can be used to engineer the junction by covalent binding^42–44^. Single-molecule devices are fabricated using a condensation reaction between molecular amine groups and carboxylic groups on the edge of the graphene nanogap to form amide bonds^44^. Chips with freshly prepared graphene nanogaps were immersed in 0.5 ml dry NEt_3_, after which 20 mg of the amide coupling reagent, hexafluorophosphate azabenzotriazole tetramethyl uronium, was added. Then, 0.5 ml of a dry CH_2_Cl_2_ solution of ZnP (0.2 mmol l^−1^) was added. The mixture was then left at room temperature in the dark for 48–72 h. After the reaction, the chip was washed with CH_2_Cl_2_ and isopropyl alcohol and blown dry with nitrogen. Then the chip containing molecular devices was connected to a chip holder via wire bonding, loaded in an Oxford Instruments 4 K PuckTester and cooled to cryogenic temperatures (4–100 K) for detailed measurements. All current (*I*_sd_) maps and traces presented in the main text and supplementary files are unprocessed. Conductance data (*G*_sd_) were calculated by applying a Savitzky–Golay filter (window length, 15; order, 5 or 7) to *I*_sd_ and then differentiating to give *G*_sd_ = *∂I*_sd_/*∂V*_sd_. Subthreshold swings (*S*_s-th_) were calculated from the steepest part of a log_10_(*I*_sd_) versus *V*_g_ trace (over two decades). The measurement was repeated six times for each temperature (see Supplementary Figs. 5-1–5-9 for the traces) and then averaged. The error reported is twice the standard deviation.

In total, we measured the current maps (*I*_sd_ versus *V*_sd_, *V*_g_) before and after the amide coupling procedure for 440 devices over six chips at room temperature. Sixteen of these devices (3.6%) showed large conductance increases and resonant transport features only after the coupling procedure, indicating that a molecular device had been formed. Most of the other devices showed either resonant transport features before molecular deposition or open-circuit behaviour after the coupling, in line with the results from *π*-stacking single-molecule graphene devices, discussed in depth in ref. ^41^. Four chips that had a molecular device were wire-bonded and loaded into an Oxford Instruments PuckTester cryostat for low-temperature measurements. All four molecular devices (devices 1–4) measured at low temperature showed a DQI dip around *V*_g_ = 0 V. Two devices (devices 1 and 2—shown in the main text) were subjected to detailed transistor characterization over a range of temperatures. The charge transport properties of devices 3 and 4 are shown in Supplementary Fig. 3-7, and the performance of all the four devices are compared in Supplementary Table 3-1.

### Theoretical calculations

Geometry optimizations were carried out using Gaussian 16 (ref. ^45^) for the isolated molecular structure using the University of Oxford Advanced Research Computing (ARC) facility, and SIESTA was used for the graphene-based junctions^46^. Transmission spectra were calculated from the Hamiltonian and overlap matrices of the DFT calculation of the junction using the GOLLUM^47^ quantum transport code. The atom-to-atom transmission pathways were obtained by using DFT combined with the non-equilibrium Green’s function method, implemented in the QuantumATK S-2021.06-SP1 software package^20^. Full details are given in Supplementary Section 8.

## Online content

Any methods, additional references, Nature Portfolio reporting summaries, source data, extended data, supplementary information, acknowledgements, peer review information; details of author contributions and competing interests; and statements of data and code availability are available at 10.1038/s41565-024-01633-1.

### Supplementary information

Supplementary Information Chemical synthesis, Fabrication images, Additional transport measurements, RC time calculation, Subthreshold swing measurement, Temperature-dependent switching, Temperature dependence of resonant tunnelling and DFT calculations.

## Data Availability

All the data supporting the findings of this study are available within the article, its Supplementary Information or from the corresponding authors upon request.
