# Supplementary material for: Quantum interference enhances the performance of single-molecule transistors
Source: Nat Nanotechnol. 2024 Mar 25;19(7):986–92. doi: 10.1038/s41565-024-01633-1 (PMC11286519; doi:10.1038/s41565-024-01633-1)
Supplement: Supplementary file 1 — Chemical synthesis, Fabrication images, Additional transport measurements, RC time calculation, Subthreshold swing measurement, Temperature-dependent switching, Temperature dependence of resonant tunnelling and DFT calculations. [file 41565_2024_1633_MOESM1_ESM.pdf]

nature nanotechnology

Article https://doi.org/10.1038/s41565-024-01633-1

Quantum interference enhances the performance of single-molecule transistors

In the format provided by the authors and unedited

Contents

Supplementary Section 1 Chemical Synthesis 2
Supplementary Section 2 Fabrication Images 6
Supplementary Section 3 Additional Transport Measurements 7
Supplementary Section 4 RC Time Calculation 11
Supplementary Section 5 Subthreshold Swing Measurement 12
Supplementary Section 6 Temperature-Dependent Switching 17
Supplementary Section 7 Temperature-Dependence of Resonant Tunnelling 18
Supplementary Section 8 DFT Calculations 19
Supplementary References 26

Supplementary Section 1 Chemical Synthesis

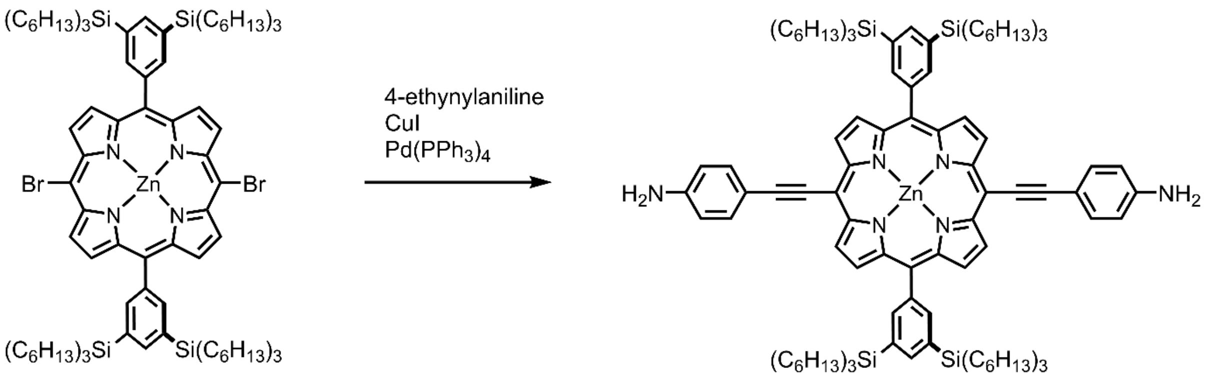

Scheme S1-1. Synthesis of **ZnP** *via* Sonogashira coupling.

General Synthetic Details

Reagents were purchased from commercial sources. Solvents were used as supplied (analytical/HPLC-grade from Fisher or Sigma-Aldrich). Petrol ether (PE) over a boiling point range of 40–60 °C was used. Column chromatography was carried out using Merck Geduran silica gel 60 under N_2 pressure. TLC was carried out on Merck silica gel 60 F254 Al plates. MALDI-TOF-MS was carried out in positive reflectron mode using a Bruker MALDI microflex instrument with dithranol as a matrix. NMR spectroscopy measurements were recorded using a Bruker AVII400 instrument. All peaks were referenced to the residual solvent peak. **Br_2ZnP** was synthesized according to a previously reported procedure,[1] and the amine anchors were added by Sonogashira coupling, as shown in Scheme S1-1.

Synthesis of ZnP: **Br_2ZnP** (25 mg, 14 µmol), 4-ethynylaniline (10 mg, 83 µmol), $Pd(PPh_3)_4$ (5 mg, 4 µmol) and CuI (2.5 mg, 14 µmol) were placed in a Schlenk tube. Toluene (3 mL) and NEt_3 (1 mL) were added and the resulting mixture was degassed and stirred for 6 h at 60 °C. The solvents were removed under reduced pressure and the crude product dissolved in CH_2Cl_2 (25 mL) and washed with brine (2 x 25 mL). The solvent was removed under reduced pressure and the crude product purified by column chromatography (PE -> CH_2Cl_2) to give 5 mg (yield: 57%) of **ZnP** as a green waxy solid.

^{1}H NMR (400 MHz, $CDCl_3$ + pyridine-d_5): δ = 9.68 (d, *J* = 4.5 Hz, 4H), 8.82 (d, *J* = 4.5 Hz, 4H), 8.24 (d, *J* = 1.1 Hz, 4H), 7.97 (t, *J* = 1.1 Hz, 2H), 7.80 (d, *J* = 8.5 Hz, 4H), 6.80 (d, *J* = 8.5 Hz, 4H), 3.89 (s(br), 4H), 1.20–1.55 (96H, m), 0.77–0.85 (m, 60H).

MALDI-TOF: m/z: 1884.8 ($C_{120}H_{182}N_6Si_4Zn$, M^+ requires 1884.3)

Supporting Characterization Data

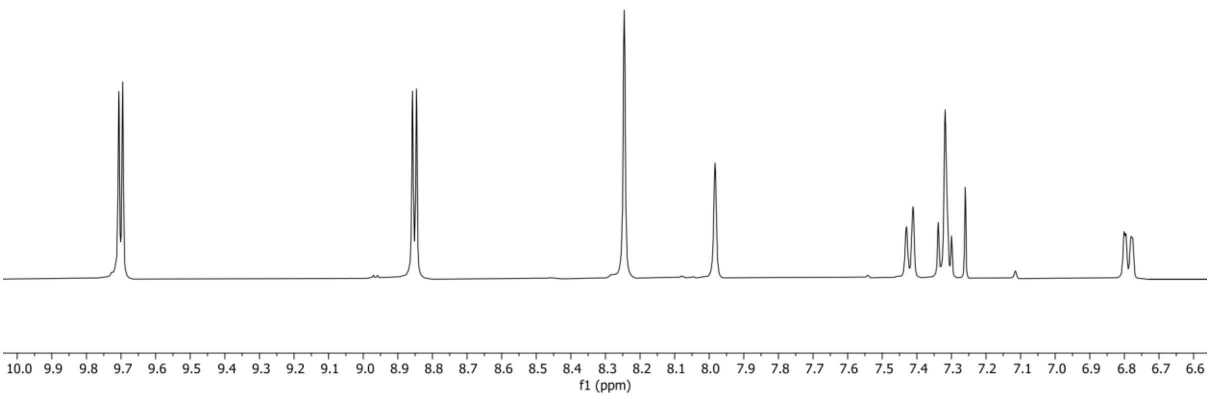

Fig. S1-1. ^{1}H NMR spectrum (400 MHz, chloroform-*d,* pyridine-*d5,* 298 K) of **ZnP**, showing the downfield region.

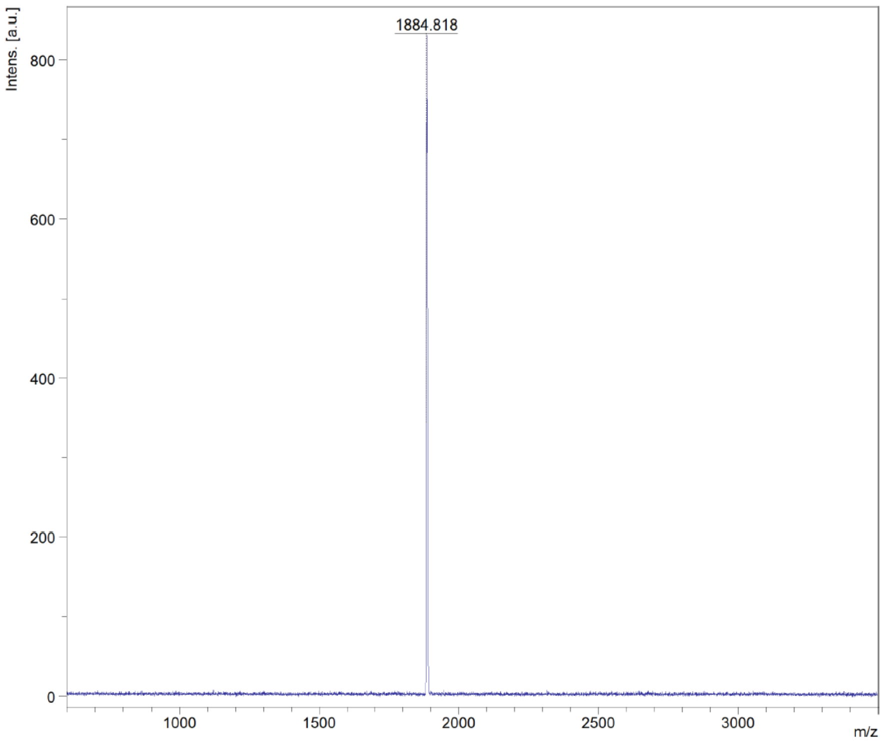

Fig. S1-2. MALDI-TOF spectrum of **ZnP** using a dithranol matrix.

Test reactions

We tested the reaction conditions we used to form molecular devices (given in Methods section) on small molecules. 9-Anthracenecarboxylic acid and phenanthrene-4-carboxylic acid were used to mimic carboxylic acid groups at zigzag and armchair graphene edges respectively, as shown in Fig. S1-3. Whilst the coupling of 9-anthracenecarboxylic acid with phenylenediamine (0.45 eq.) gave the diamide in a 45% yield, the reaction with phenathrene-4-carboxylic acid, monitored by TLC, showed no conversion after 48 hours. We attribute the lack of reactivity to steric hindrance around the carboxylic acid group, and conclude that a molecular junction is unlikely to form at a locally armchair graphene edges.

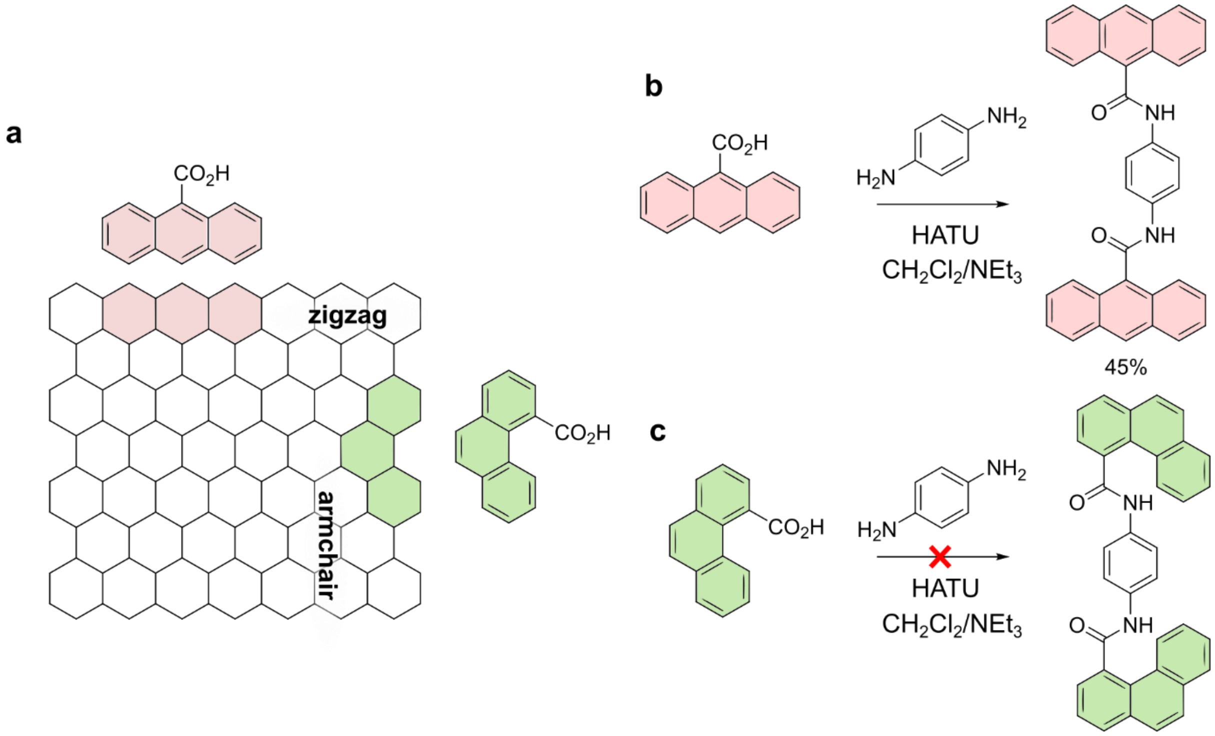

Fig. S1-3. a 9-Anthracenecarboxylic acid (red) and phenanthrene-4-carboxylic acid (green) as model compounds for carboxylic acid terminations at zigzag and armchair graphene respectively. **b**, **c** Reaction schemes for amide coupling of the acids with phenylenediamine (0.45 eq.). Hexafluorophosphate azabenzotriazole tetramethyl uronium (HATU) is used as an amide-coupling reagent. The solvent is a 1:1 mixture of dichloromethane and triethylamine. These are the same conditions as for the molecular devices.

Synthesis of *N,N′*-(1,4-phenylene)*bis*(anthracene-9-carboxamide):

9-Anthracene carboxylic acid (55.6 mg, 0.25 mmol), HATU (200 mg, 0.5 mmol), and 1,4-phenylene diamine (11 mg, 0.11 mmol) were dissolved in a 1:1 mixture of NEt_3:CH_2Cl_2 (17 mL). The reaction was followed by TLC. After 16 hours, the reaction mixture was washed with brine (2 x 25 mL) and $NaHCO_3$ (aq.) (2 x 25 mL). The solvent was removed under reduced pressure and the crude product purified by column chromatography to give 25.6 mg (yield: 45%) of *N*,*N′*-(1,4-phenylene)*bis*(anthracene-9-carboxamide) as a light yellow solid.

^{1}H NMR (400 MHz, $CDCl_3$): δ 8.89 (dd, *J* = 4.5, 1.4 Hz, 2H), 8.73 (s, 2H), 8.61 (dq, *J* = 8.8, 1.0 Hz, 4H), 8.52 (dd, *J* = 8.4, 1.4 Hz, 2H), 8.12 (ddd, *J* = 8.5, 1.6, 0.9 Hz, 4H), 7.75 (ddd, *J* = 8.8, 6.6, 1.3 Hz, 4H), 7.61 (ddd, *J* = 8.5, 6.6, 1.1 Hz, 4H), 7.53 (dd, *J* = 8.4, 4.5 Hz, 2H).

Supporting Characterization Data

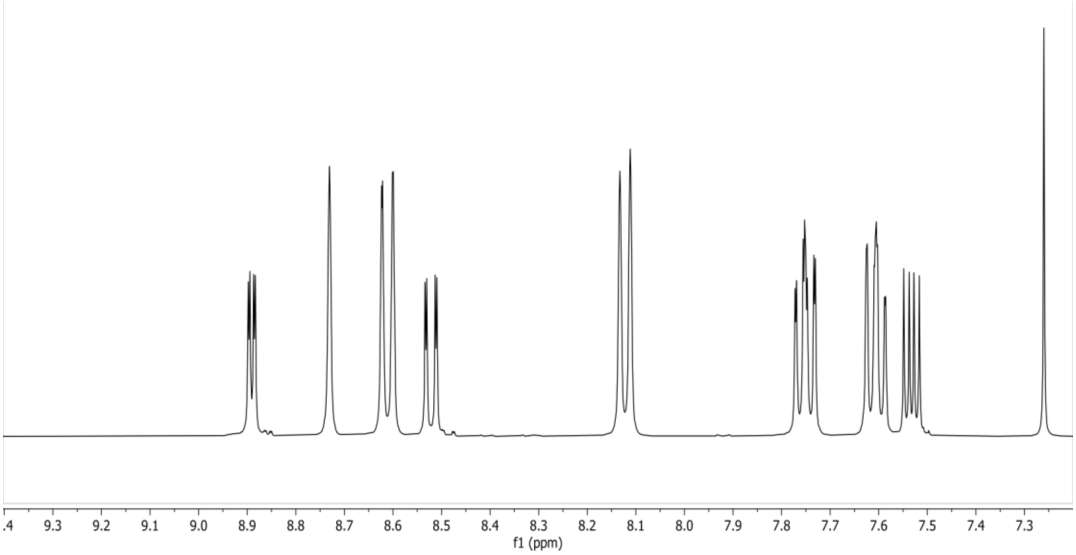

Fig. S1-4. ^{1}H NMR spectrum (400 MHz, chloroform-*d,* 298 K) of *N*,*N′*-(1,4-phenylene)*bis*(anthracene-9-carboxamide) showing the downfield region.

Supplementary Section 2 Fabrication Images

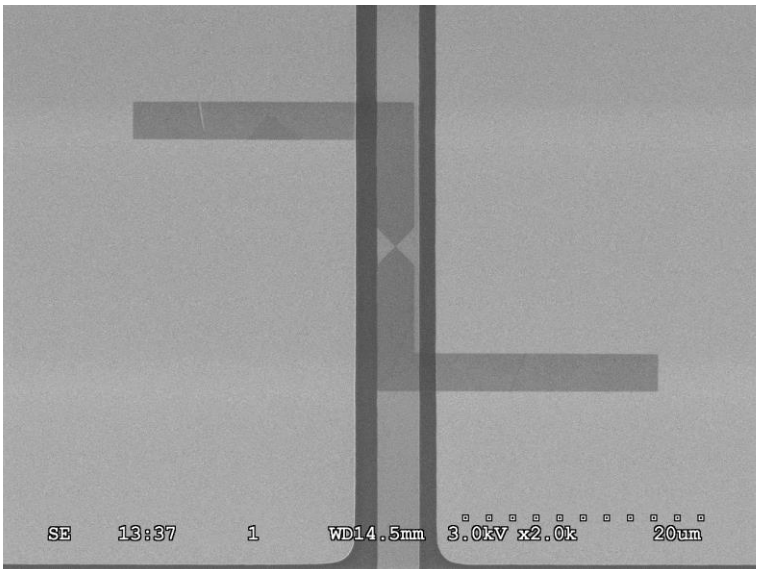

Fig. S2-1. Example SEM image of an aluminium-protected device.

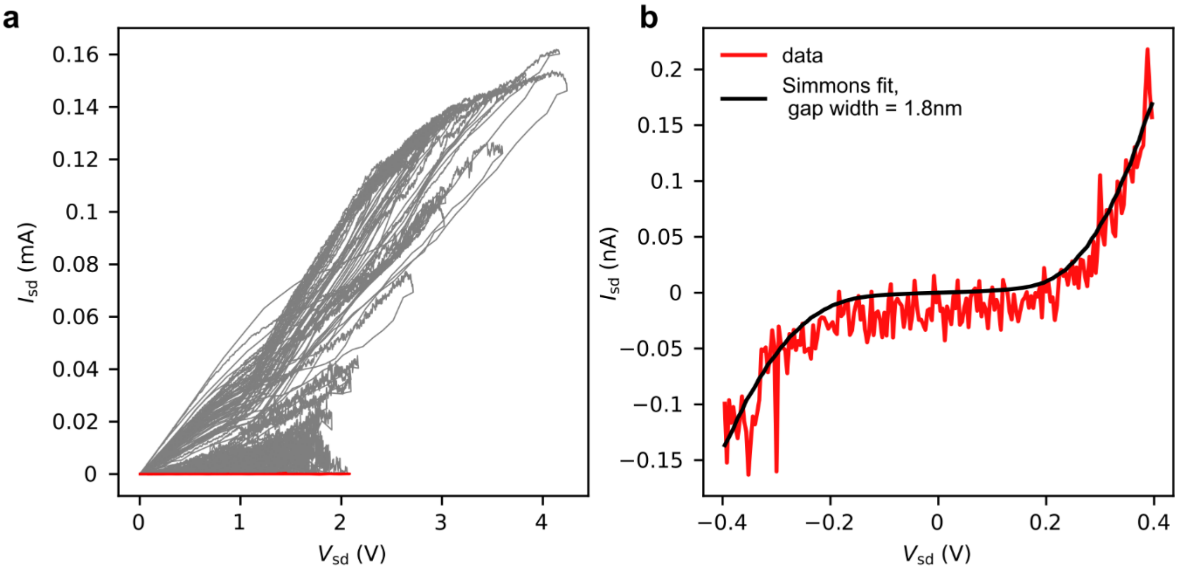

Fig. S2-2. a Feedback-controlled electroburning traces (last trace shown in red) to form the graphene nanogap of the device in the main text. **b** I_{sd}-V_{sd} curve of the same device, with a fit to the Simmon's model for an asymmetric barrier (black line).[2]

Supplementary Section 3 Additional Transport Measurements

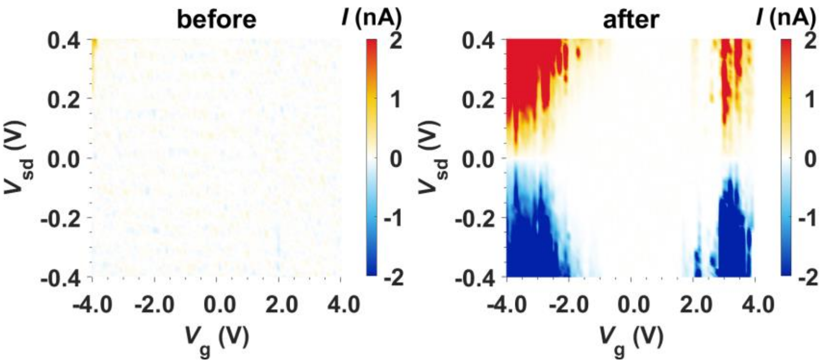

Fig. S3-1. Current map (I) as a function of bias voltage (V_{sd}) and gate voltage (V_g) before (left panel) and after (right panel) molecular connection, measured at room temperature, for the device shown in main text.

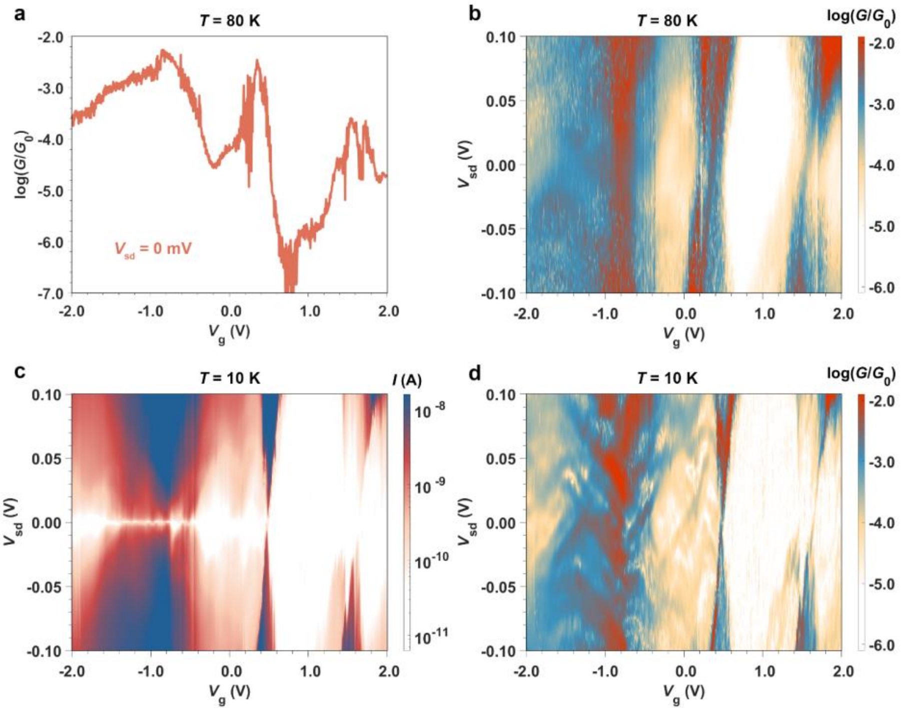

Fig. S3-2. Additional data for the device shown in main text. Zero-bias differential conductance trace (**a**) and conductance map (**b**) for device at 80 K across the full measured gate range. **c** Current map and **d** differential conductance at 10 K. Fabry-Pérot interference patterns[3] are observed at negative V_g, which disappear at 80 K ($k_B T$ = 6.9 meV) when the thermal energy is larger than the FP spacing.

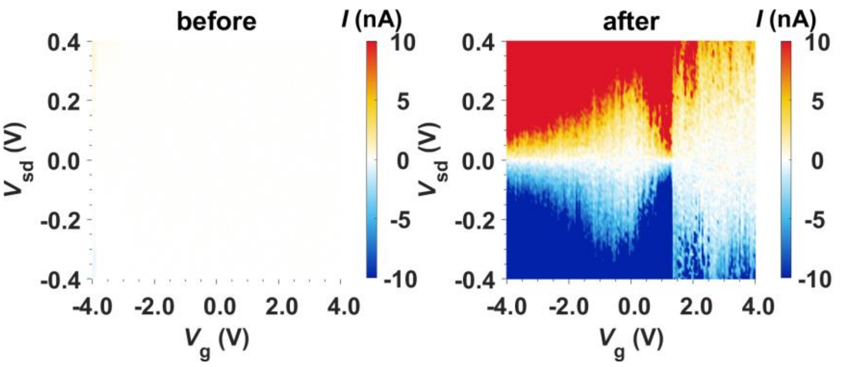

Fig. S3-3. Current map (I_{sd}) as a function of bias voltage (V_{sd}) and gate voltage (V_g) before (left panel) and after (right panel) molecular connection, measured at room temperature, for a second device (device 2) shown in Fig. S3-4 and main text Fig. 3c.

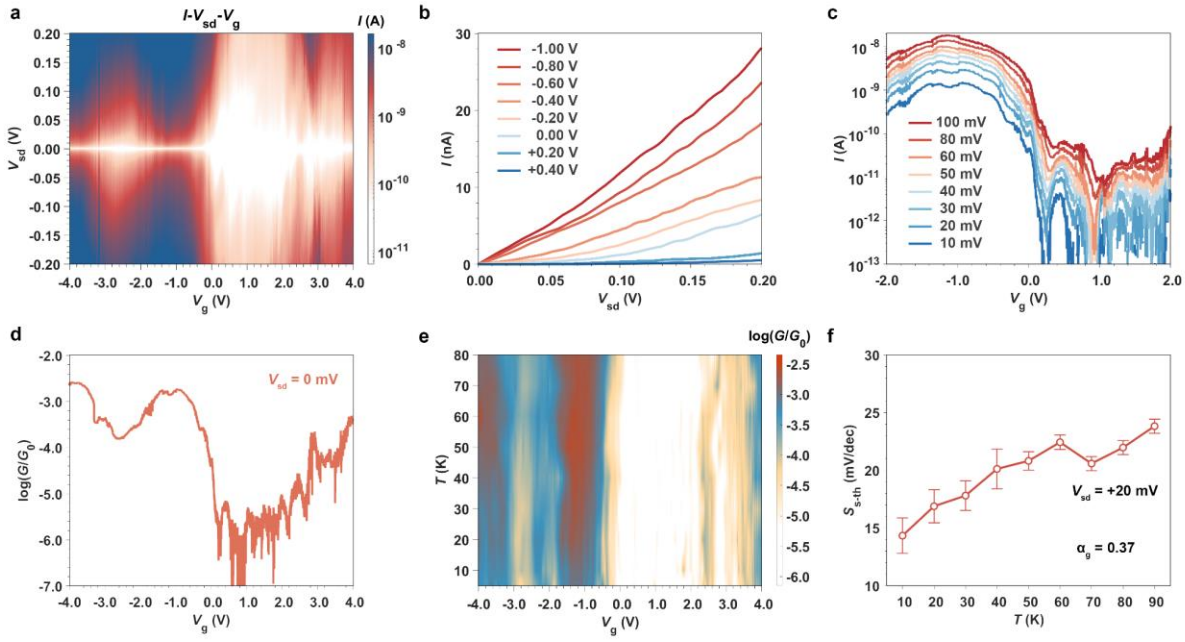

Fig. S3-4. Full transistor characterisation of device 2 shown in Fig. 3c. **a** Current map measured as a function of bias voltage (V_{sd}) and gate voltage (V_g). **b** Output characteristics and **c** transfer characteristics for the device. **d** Differential conductance measured as a function of V_g (red curve) at V_{sd} = 0 mV. The conductance is plotted in logarithmic scale as the ratio to conductance quantum, G_0. **e** Differential conductance map measured as a function of temperature and gate voltage. **f** Normalised subthreshold swing at V_{sd} = 20 mV as a function of temperature. Current map and differential conductance map measured at lower temperature can be found in Fig. S3-5. The measurement was repeated six times for each temperature and then averaged. The error reported is twice the standard deviation.

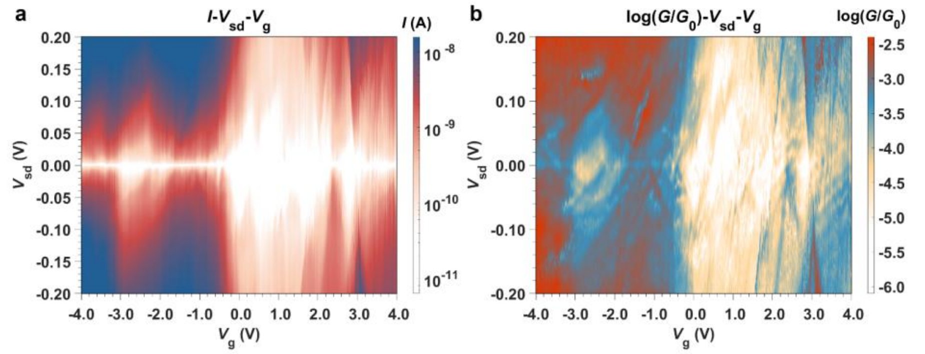

Fig. S3-5. a Current and **b** differential conductance ($G = dI_{sd}/dV_{sd}$) maps measured at 2.8 K for the device shown in Fig. S3-4.

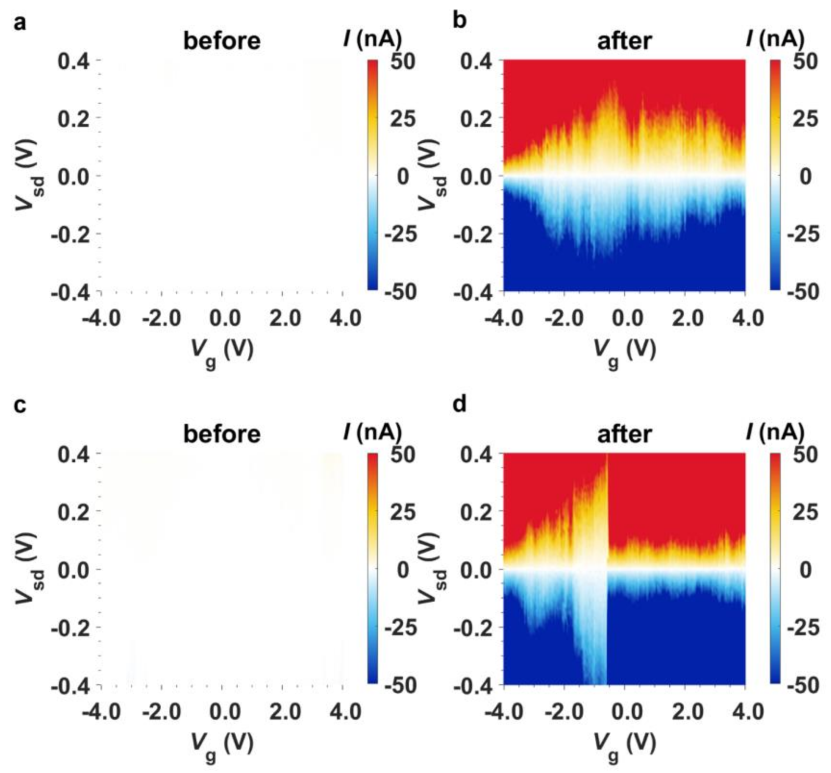

Fig. S3-6. Current map (I_{sd}) as a function of bias voltage (V_{sd}) and gate voltage (V_g) before (**a, c**) and after (**b, d**) molecular connection for devices 3 and 4, respectively, at room temperature.

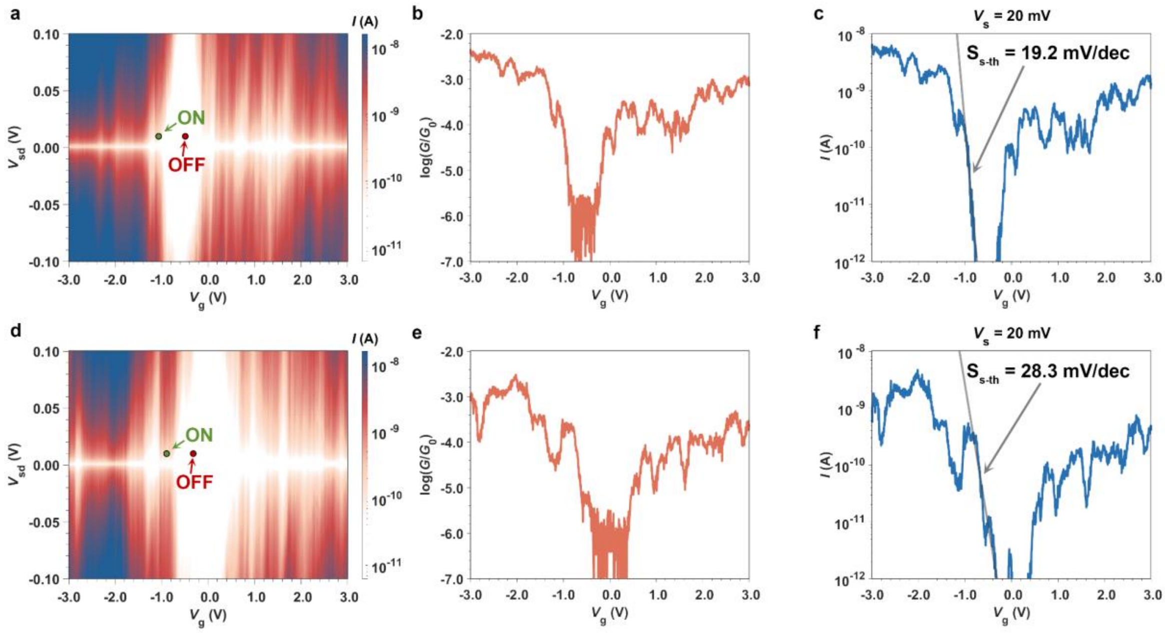

Fig. S3-7. Current maps, zero-bias conductance traces and current traces (I_{sd}-V_g at V_{sd} = 20 mV) for devices 3 (**a–c**) and 4 (**d–f**). The subthreshold swings are calculated from the reciprocal of the gradients of the grey lines. Data taken at 80 K.

Table S3-1. ON and OFF conductance (in terms of the conductance quantum, G_0), ratio, gate couplings and normalized subthreshold swings ($S_{s\text{-}th}$) for devices 1–4. Data taken at 80 K.

	G_{on} (G_0)	G_{off} (G_0)	ON/OFF ratio	α_g	$S_{s\text{-}th}$ (mV/dec)
**Device 1 - main text**	$10^{-2.73\pm0.02}$	$10^{-6.22\pm0.16}$	3.1×10^3	0.38	14.5
**Device 2- main text (Fig. 3c)/ Supplementary Materials (Fig. S3-4)**	$10^{-2.75\pm0.02}$	$10^{-6.42\pm0.57}$	4.6×10^3	0.37	22.0
**Device 3 - Supplementary Materials (Fig. S3-7)**	$10^{-2.81\pm0.04}$	$10^{-6.55\pm0.44}$	5.5×10^3	0.18	19.2
**Device 4 - Supplementary Materials (Fig. S3-7)**	$10^{-3.50\pm0.05}$	$10^{-6.70\pm0.34}$	1.6×10^3	0.14	28.3

Supplementary Section 4 RC Time Calculation

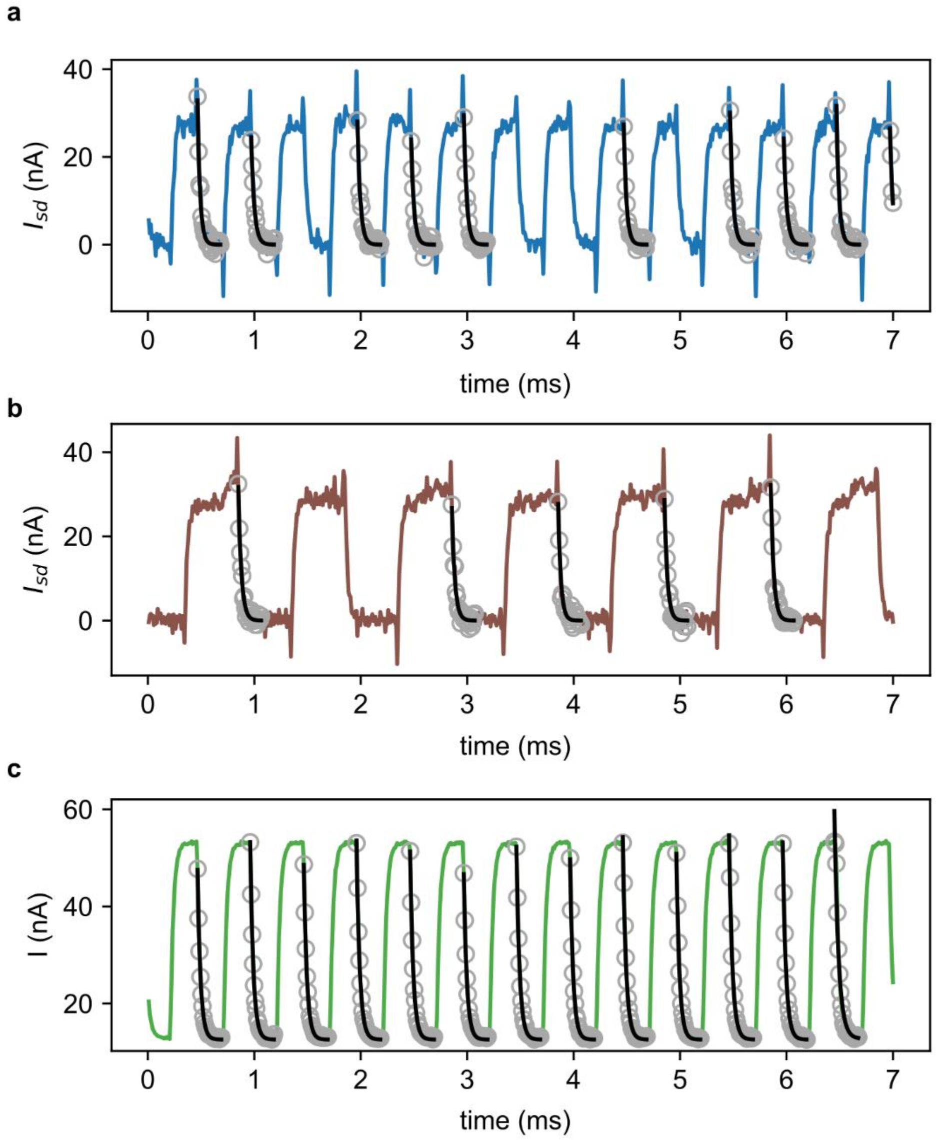

Fig. S4-1. Time-trace data. A section of switching cycles of the main-text device whilst a square-wave ($V_{g,min}$ = 610 mV, $V_{g,max}$ = 760 mV) at **a** 2 kHz, and **b** 1 kHz, is applied to the gate electrode; V_{sd} = 100 mV. The data points in grey are those fitted to the function: $I = I_0 \exp - t/RC$ (black lines) to give an RC time of 30 ± 1 μs (reported as mean ± standard deviation of 10 fitted values). As shown in (**c**), the bandwidth of the amplifier (SRS SR570, sensitivity: 10^{-7} A/V) limits the response time of the circuit, shown by bypassing the single-molecule device and applying a 2 kHz square wave ($V_{g,min}$ = 0 mV, $V_{g,max}$ = 400 mV) across a 100 MΩ resistor, which gives a very similar RC time (29 ±1 μs).

Supplementary Section 5 Subthreshold Swing Measurement

The details for subthreshold swing measurement for the devices shown in main text are described here. The bias voltage (V_{sd}) is fixed at 20 mV. At each temperature, current (I_{sd}) is measured as a function of gate voltage (V_g) six times and plotted below. The experimental values shown in Fig. 5c are the average values from the six curves. The ranges to calculate subthreshold swing are highlighted in green, in which the blue and red spots indicate the lowest and highest V_g the ranges.

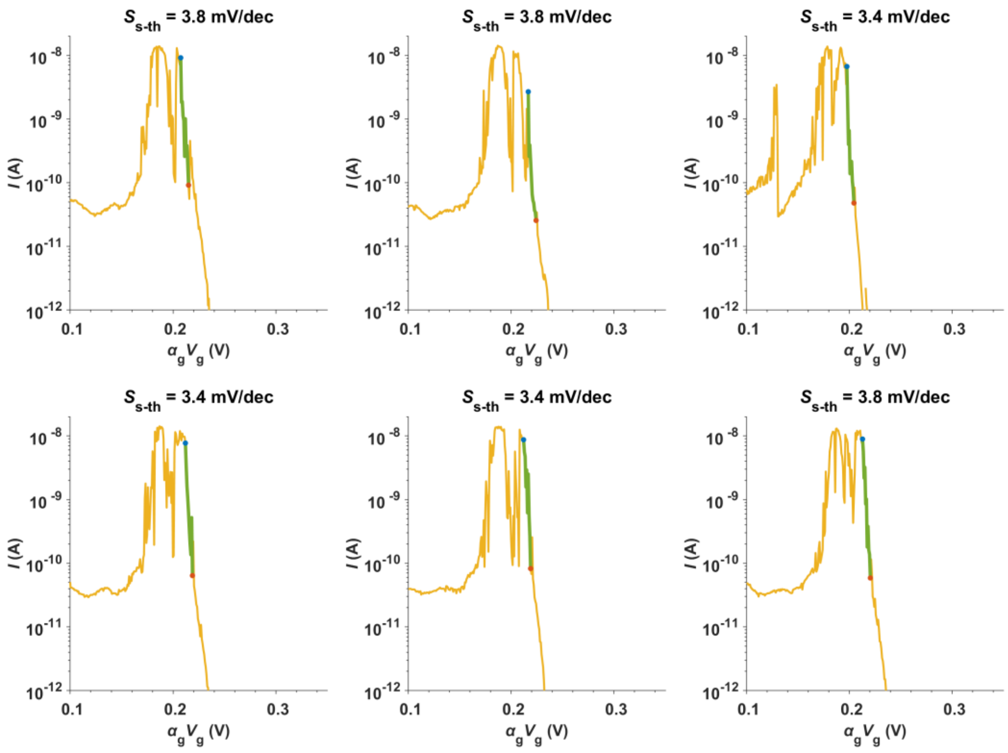

Fig. S5-1. Device 1 subthreshold swing calculation at 10 K.

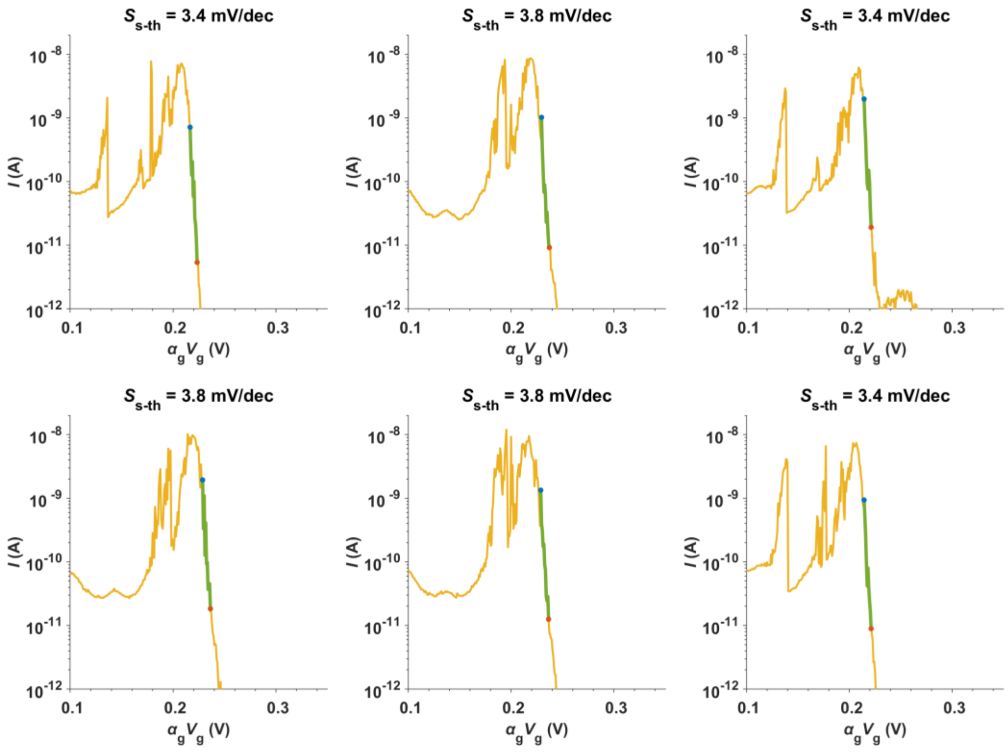

Fig. S5-2. Device 1 subthreshold swing calculation at 20 K.

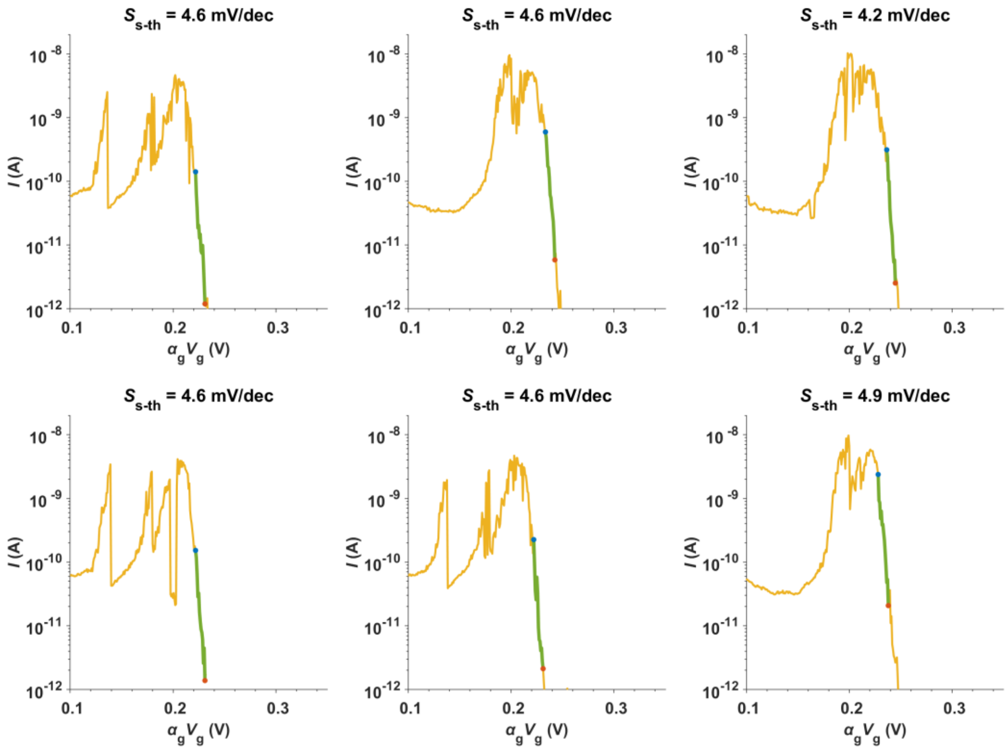

Fig. S5-3. Device 1 subthreshold swing calculation at 30 K.

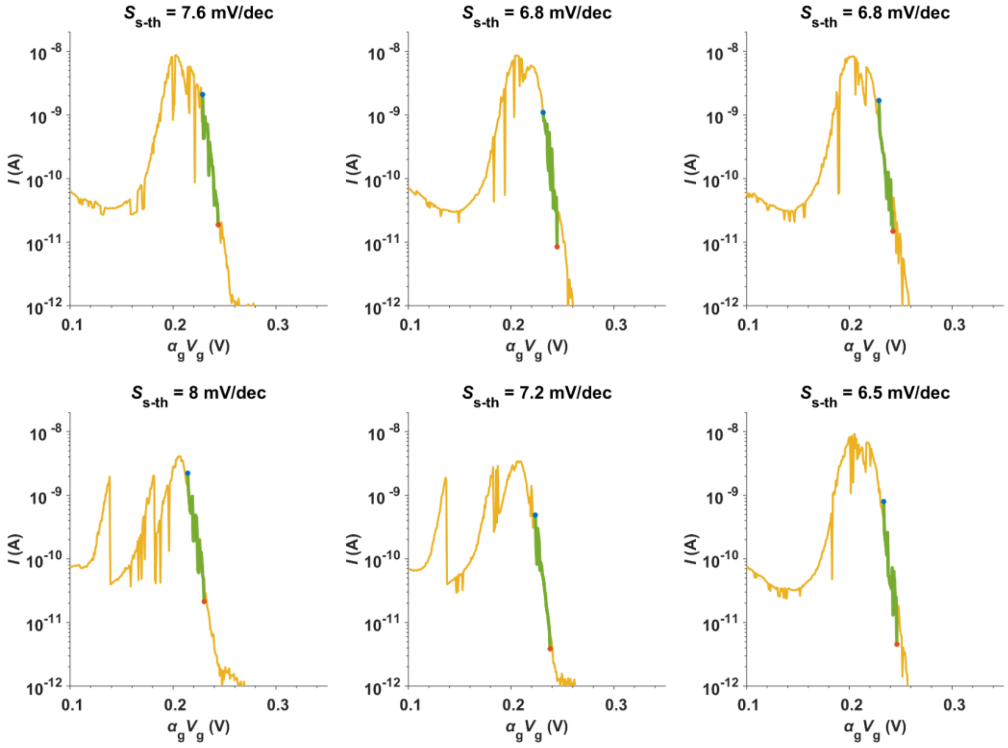

Fig. S5-4. Device 1 subthreshold swing calculation at 40 K.

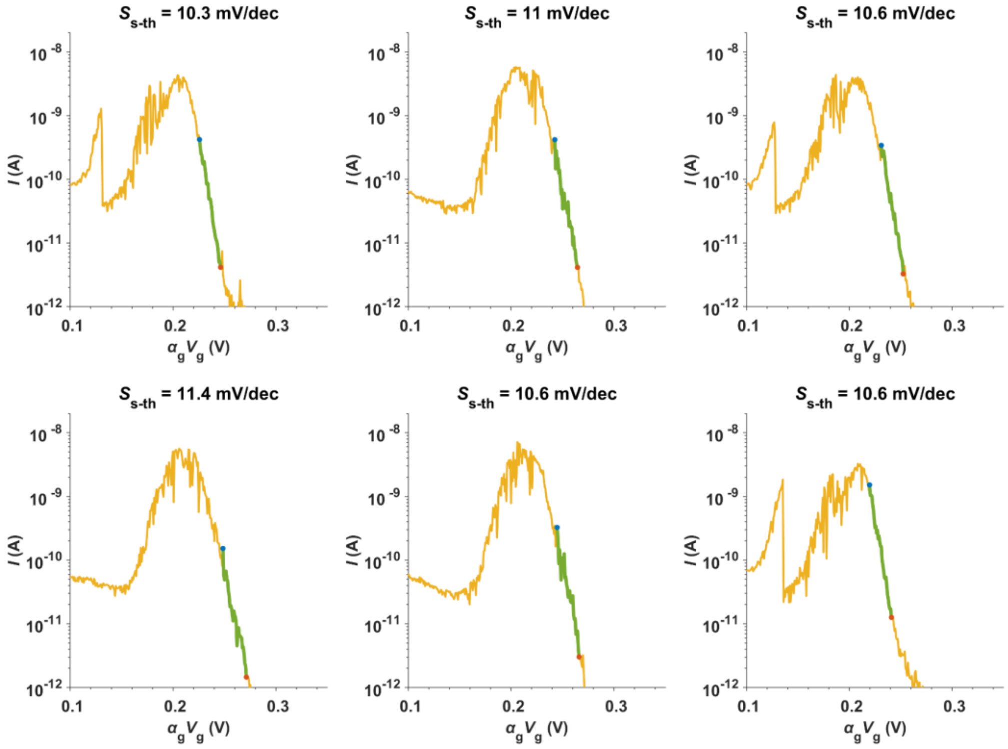

Fig. S5-5. Device 1 subthreshold swing calculation at 50 K.

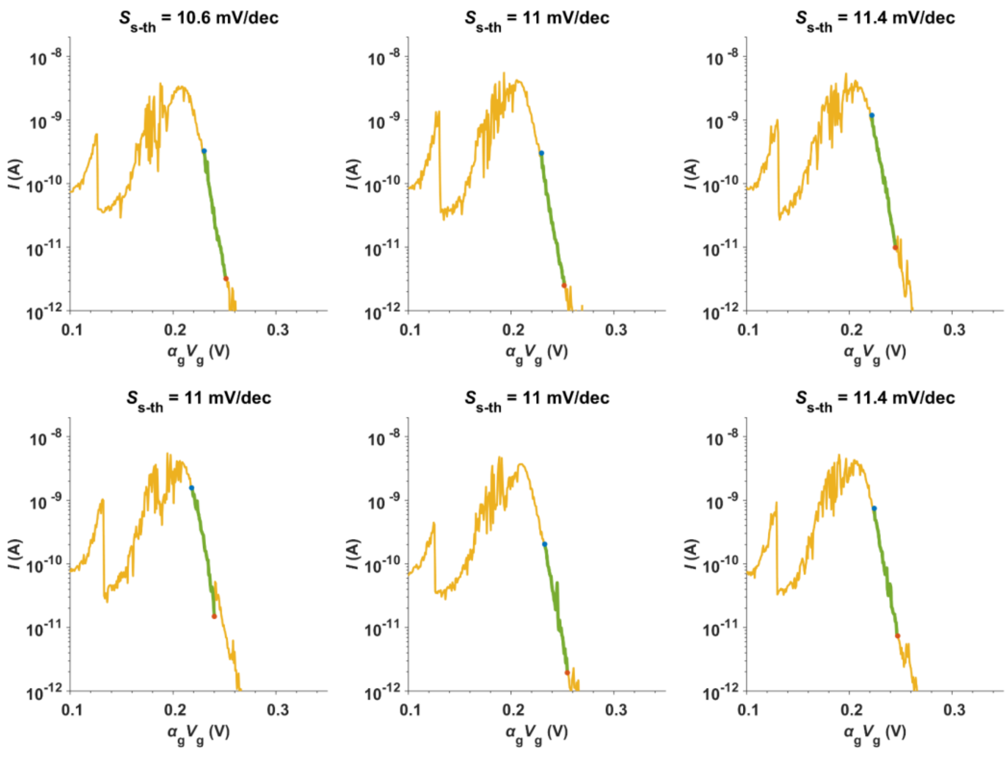

Fig. S5-6 Device 1 subthreshold swing calculation at 60 K.

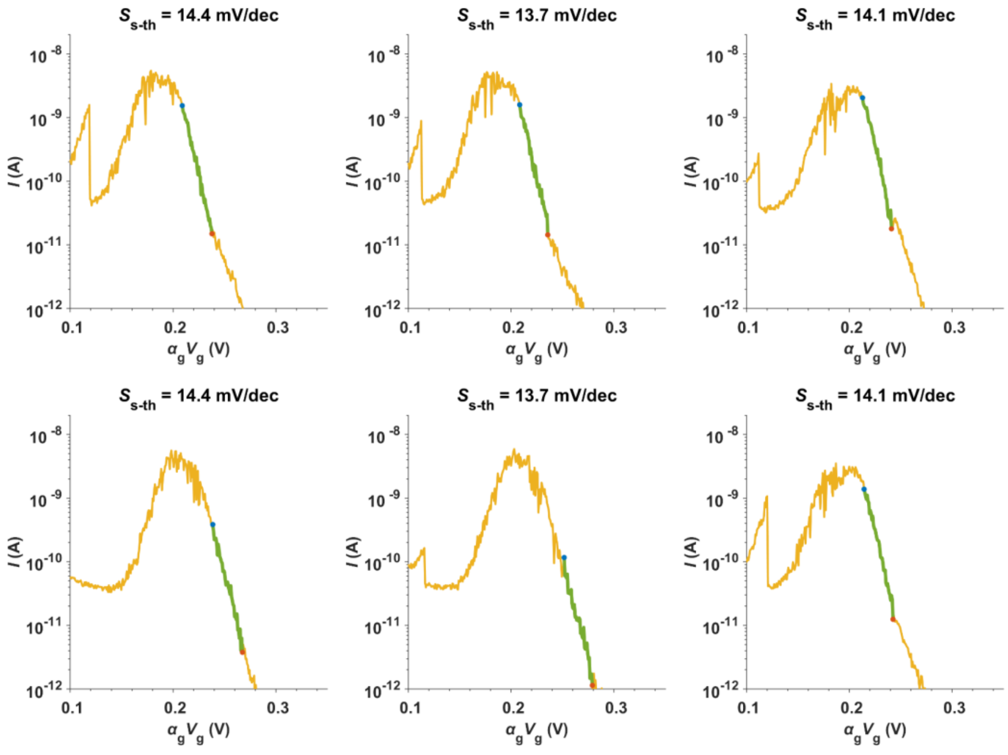

Fig. S5-7. Device 1 subthreshold swing calculation at 70 K.

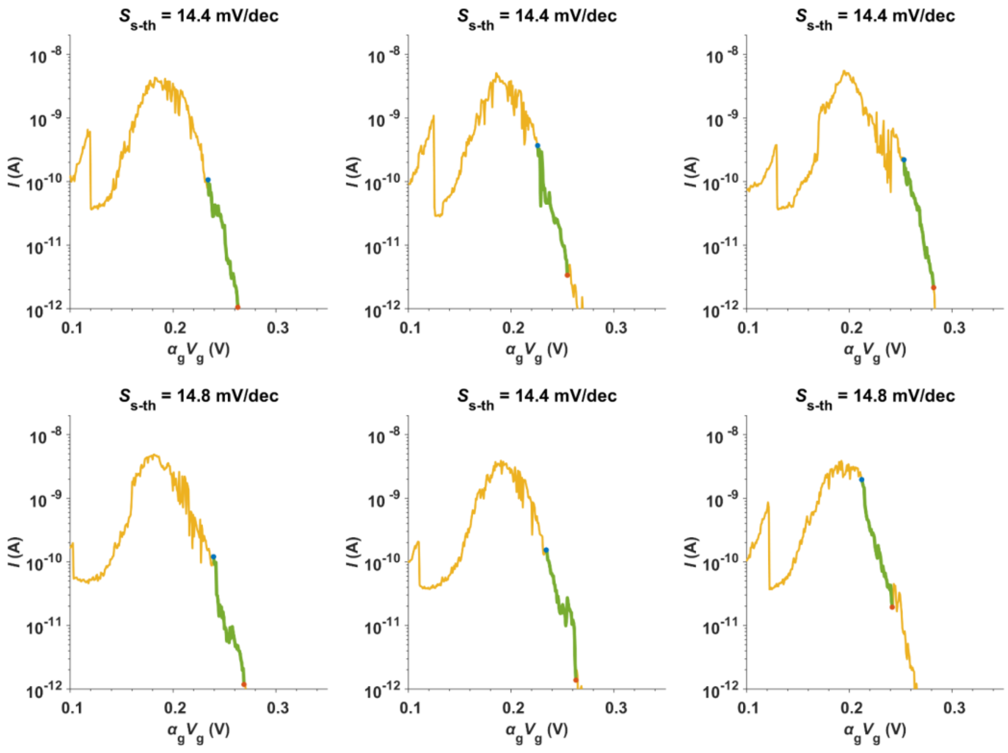

Fig. S5-8. Device 1 subthreshold swing calculation at 80 K.

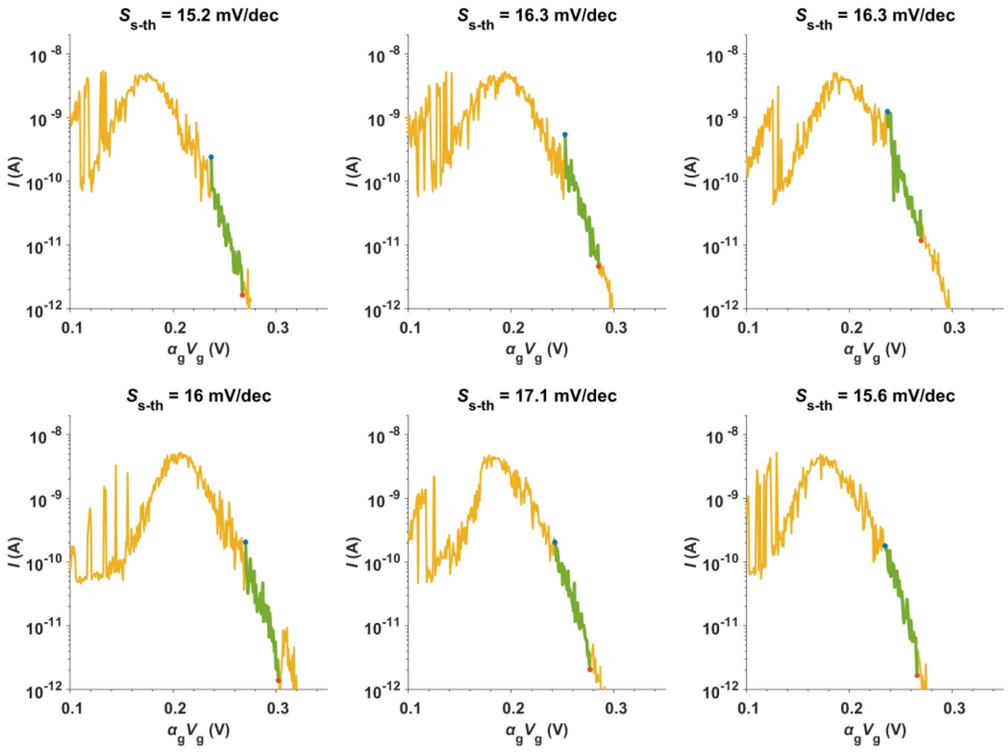

Fig. S5-9. Device 1 subthreshold swing calculation at 100 K.

Supplementary Section 6   Temperature-Dependent Switching

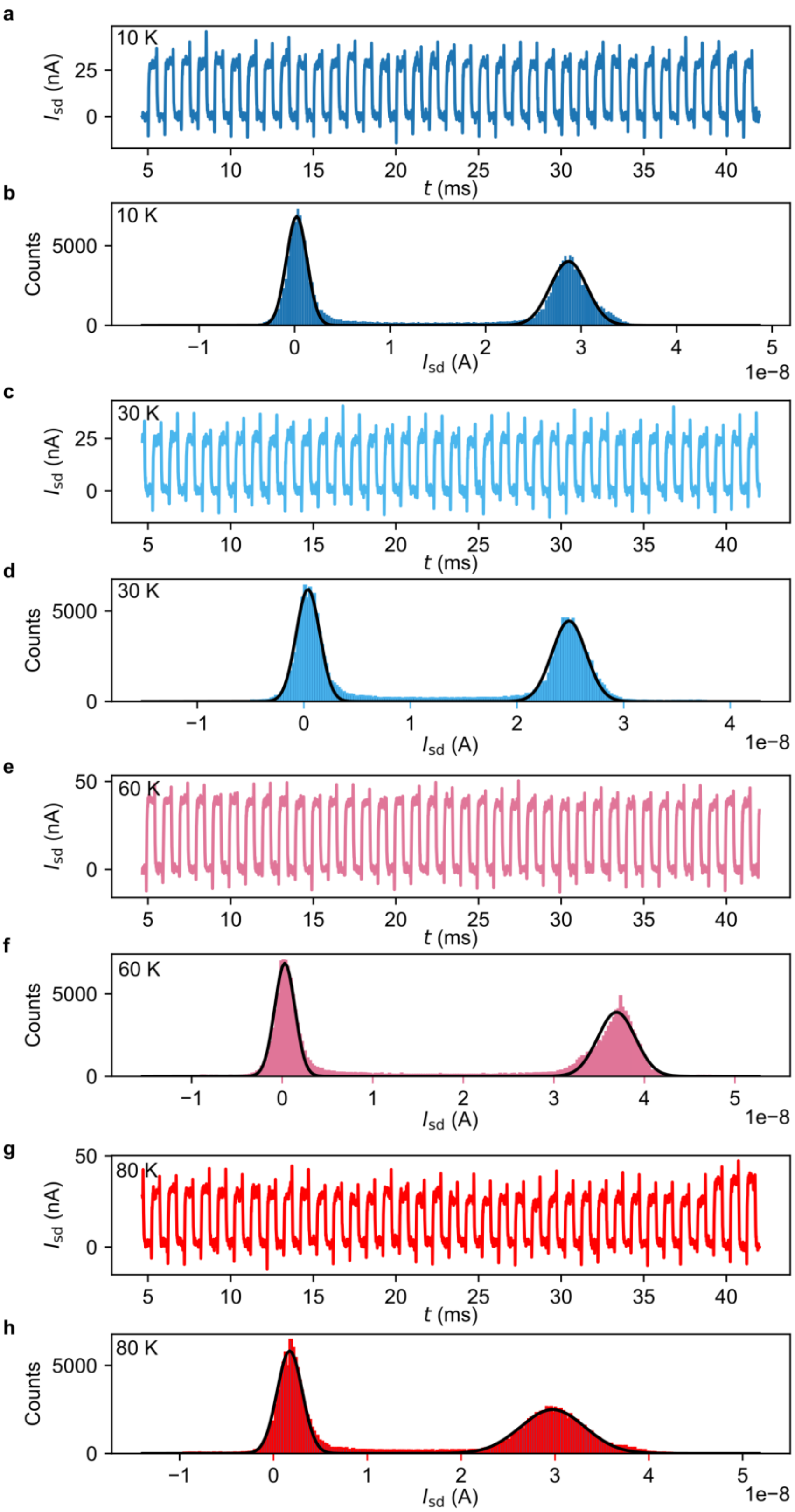

Fig. S6-1. Sections of I_{sd}(t) traces whilst a square-wave is applied to the gate electrode (2 kHz, $V_{g,min}$ = 610 mV, $V_{g,max}$ = 760 mV, V_{sd} = 100 mV) at 10 K (**a**), 30 K (**c**), 60 K (**e**), 80 K (**g**). Each full time trace is 1.6 seconds long (total cycles/trace = 3,200), and conversion to histograms show the two states at 10 K (**b**), 30 K (**d**), 60 K (**f**), 80 K (**h**).

Supplementary Section 7 Temperature-Dependence of Resonant Tunnelling

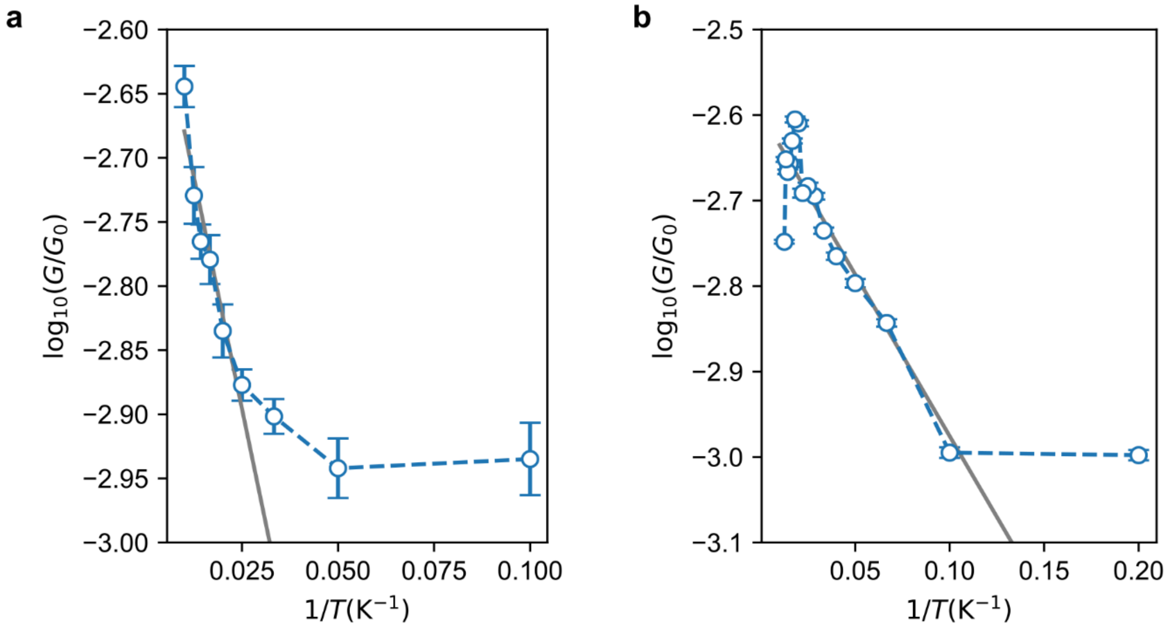

Fig. S7-1. The temperature dependence behaviour of the zero-bias conductance, G(V_{sd} = 0), on the *N*–1/*N* resonance for the device in the main text (**a**) and the second device, Fig. 3c, Fig. S3-4 (**b**). Both devices have a temperature-independent conductance at low temperature, but as temperature is increased above 20 K (**a**) or 10 K (**b**), the conductance increases with a roughly linear (grey) log(G/G_0) vs T^{-1} relationship. The conductance value is obtained from fitting the *N*–1/*N* resonance peak to a thermally broadened function,[4] and the error bar is the standard deviation of the peak height.

Supplementary Section 8 DFT Calculations

Theoretical method and parameters

The isolated molecular orbitals (Fig. S8-1) were output from a geometry optimization calculation using a B3LYP functional and a basis set combination of 6-31g* for H, C, N, O and LanL2DZ pseudopotential for Zn, in Gaussian 16.[5] The visualizations (obtained from the relevant cube files) of MOs in Fig. 2c and Fig. S8-1 were made in Multiwfn[6] and VMD[7].

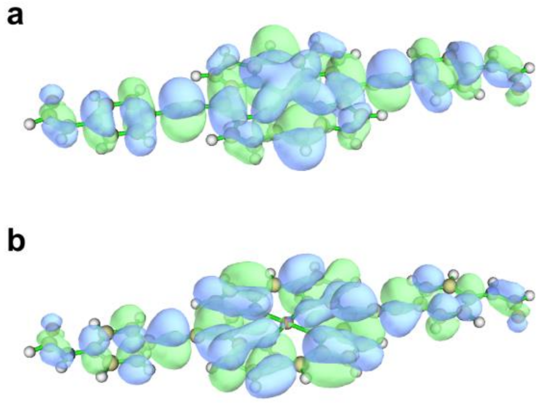

Fig. S8-1. (**a**) LUMO and (**b**) HOMO of **ZnP** with terminal amine groups (-NH_2) converted to amides (-NHC(O)H) so orbital coefficients on the connecting carbon atoms are visualized. The coefficients of the MOs on the connecting carbon atoms are (+, +) for the LUMO (left, right) and (+, –) for the HOMO, with green: '+' and blue: '–', a configuration not consistent with observation of DQI in the HOMO/LUMO gap.[8] The solubilizing groups on the lateral *meso* positions of the porphyrin were replaced with H atoms to reduce the size of the calculations.

Fig. S8-1 rules out the isolated MOs as the source of DQI, so we proceeded to perform LDOS and transport calculations on the full system. The graphene-based junction was constructed and density functional calculations were performed using the SIESTA code.[9] The LDA approximation[10] was used to describe the exchange correlation functional. A mesh cut-off of 150 Rydbergs was used to determine the fineness of the real space grid and a single zeta basis set was chosen. The radius confinement of the orbitals was defined by an energy shift of 0.001 Rydbergs and norm-conserving pseudopotentials were used. Again, the solubilizing groups on the lateral *meso* positions of the porphyrin have been replaced with H atoms to reduce the size of the calculations and the molecule was attached to the graphene electrodes each consisting of 120 carbon atoms. The edge atoms of the graphene were passivated by hydrogen atoms. The molecule and edge carbon and hydrogen atoms were allowed to relax until all forces on the atoms were less than 0.01 V/Å, the remaining atoms in the graphene electrodes are fixed. The electronic Hamiltonian was then extracted, and the zero-bias transmission coefficient T(E) was calculated using the equilibrium transport code GOLLUM.[11]

Graphene electrode local density of states

Preliminary calculations showed that the DQI dip was present when the molecule was connected to a zigzag wedge edge and absent when the molecule was connected to a graphene nanogap with armchair edges. Therefore, we studied different shapes of the graphene edges. The local density of states (LDOS) for three graphene edges were calculated (Fig. S8-2). The flat edge (W=0) has an armchair structure. By adding carbon atoms to the armchair edge a wedge, or protrusion, can be generated with two zigzag edges of length, W, similar to previous computational studies.[12,13] The LDOS is evaluated at the Fermi energy and plotted for each of these structures and shown in Fig. S8-2. The weighting of the LDOS on the

edge carbon atoms is constant for the flat W = 0 edge. As the size of the wedge is increased the value of the local density of states on the terminal carbon atoms decreases for W = 3 and decreases further for W = 5. The weighting of the LDOS on the edge carbon atoms not at the apex of the wedge remains large.

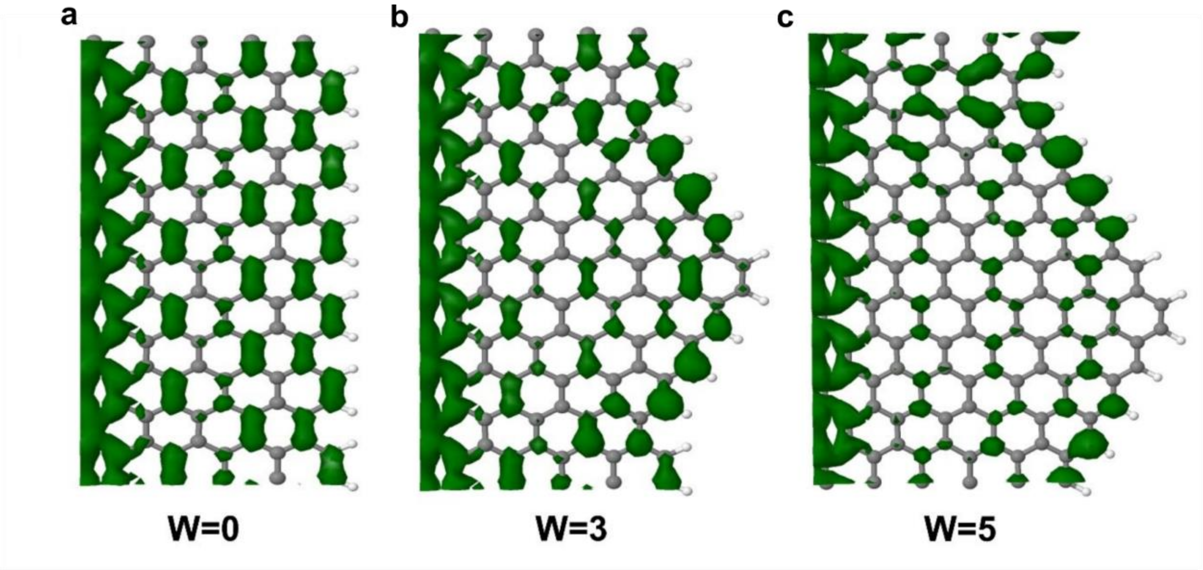

Fig. S8-2 LDOS for graphene edges of different sizes W = 0 (**a**), W = 3 (**b**) and W = 5 (**c**)

Transmission coefficient

For modelling the molecular junction, the results in Fig. S1-3 show that graphene electrodes with –COOH groups at W = 0 edges are unreactive to amide coupling chemistry, so W = 5 was chosen, and the porphyrin was attached at the terminating edge. The relaxed junction geometries are shown in Fig. S8-3. Two different isomers of the anchor groups were investigated, the amide connection (NHCO, Fig. S8-4) and an enol (NCOH, Fig. S8-5) connection. Whilst the amide connection will be present in the experimental devices, we studied the enol connection to rule out cross-conjugation (only present in the amide links) as the source of destructive quantum interference.[14] We calculated the LDOS at the Fermi energy, the projected density of states on the connecting carbon atom of the graphene electrode, and the transmission coefficient *T*(E). We also changed the position of contact between the molecule and the graphene so that four different locations are calculated.

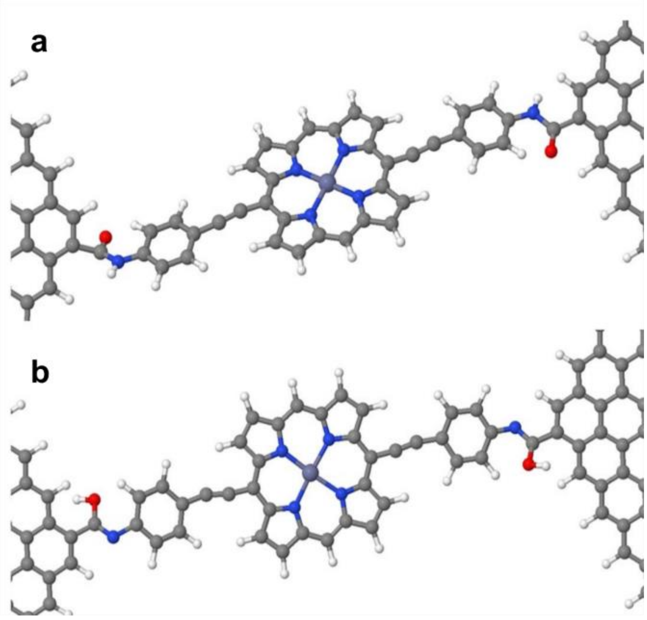

Fig. S8-3 Relaxed junction geometry for the molecules attached to the edge of W = 5 graphene electrodes for two different anchor groups (**a**) NHCO and (**b**) NCOH.

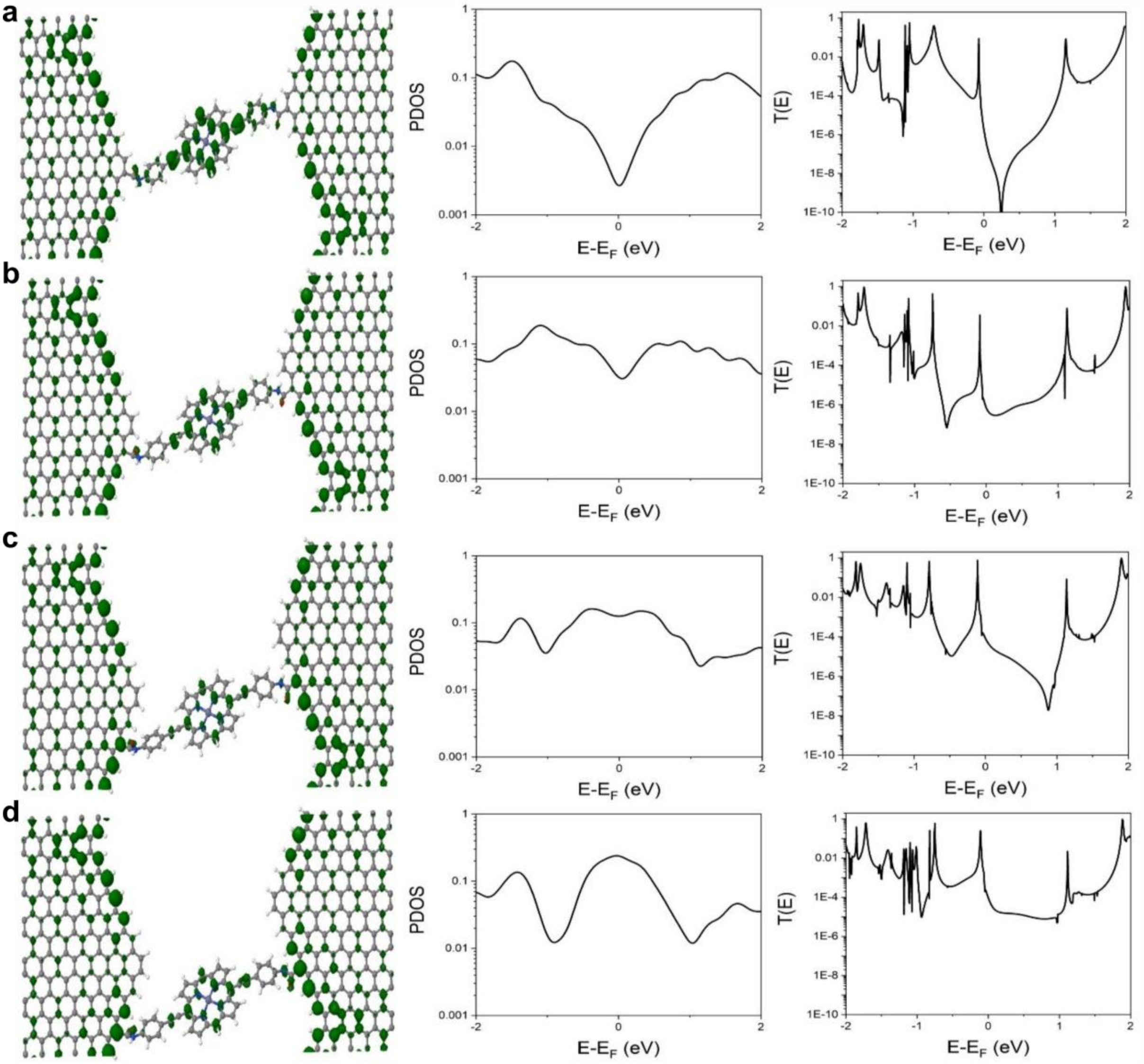

Fig. S8-4 NHCO molecular junction for different contact locations along the edge of W = 5 graphene electrodes. (Left panel) LDOS at the Fermi energy for geometries **a**-**d**. (Middle panel) PDOS of the contacting carbon atom of the graphene. (Right panel) Zero bias transmission coefficient *T*(E) as a function of electron energy.

For the NHCO molecular junction, the transmission of the Fig. S8-4a geometry shows that there is a dip in *T*(E) close to the Fermi energy. This coincides with a minimum in the projected density of states PDOS, and the LDOS shows no weight on the contacting carbon atom. As the contact site is shifted along the edge, for the geometry in Fig. S8-4b, the dip in the PDOS at the Fermi energy is decreased which corresponds to the dip in the transmission *T*(E) being smaller. For the geometry in Fig. S8-4c, the local density of states at the Fermi energy is large at the contacting carbon atom and therefore the PDOS is close to a maximum. The transmission value shows no dip at the Fermi energy. This behaviour is repeated for the geometry in S8-4d.

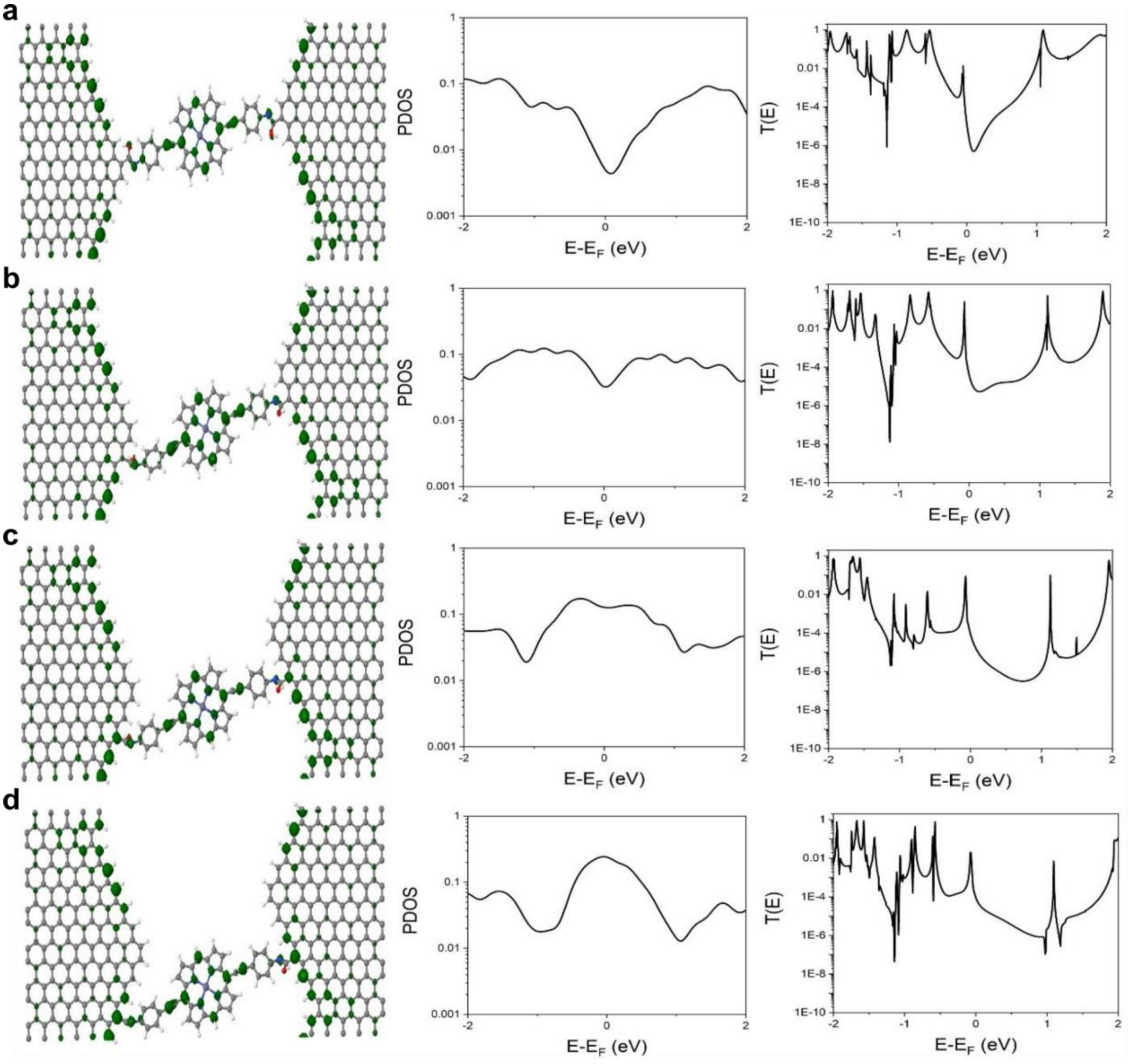

Fig. S8-5 NCOH molecular junction for different contact locations along the edge of W=5 graphene electrodes. (Left panel) LDOS at the Fermi energy for geometries **a-d**. (Middle panel) PDOS of the contacting carbon atom of the graphene. (Right panel) Zero bias transmission coefficient *T*(E) as a function of electron energy.

Repeating the calculations for the NCOH anchor group in (Fig S8-5) shows similar behaviour to the NHCO connection, with transmission dips occurring when the molecule is attached to the end of the wedge. Therefore, we conclude that the current and conductance suppression in the *N* state shown in the experimental data is not due to interference features defined by the molecular orbitals, nor cross-conjugation in the amide links, but rather the edge structure at the molecule-graphene interface. This behaviour has been predicted previously in a computational study of graphene-based single-molecule devices.[12]

We display a schematic of the graphene-molecule interface origin of the interference condition in Fig. S8-6. For the case of a generic wedge shape, the transmission coefficients of graphene edge states (which dominate charge transport due to their proximity to the Fermi level of undoped graphene) interfere at the terminal atom and can create standing waves with their reflections, suppressing molecular transmission, as shown in Fig. S8-6a.

For the specific wedge topology shown in Fig. 2 and Fig. S8-2 to S8-4, zigzag edges merge into a locally armchair edge (highlighted in magenta). The wavefunctions at the Fermi energy of undoped graphene have a wavelength of $2a_0$ (a_0 being the magnitude of the lattice parameter) with nodes (antinodes) on the B (A) atoms for t_1 and vice versa for t_2. Therefore, when t_1 and t_2 reach A* /B* sites they can no longer propagate around the edge (or into free space) and undergo reflection, as shown in Fig. S8-6b. The interference of the transmitted/reflected waves creates a standing wave with A* and B* as nodes. The result shown from the DFT-calculated DOS in Fig. S8-2 is consistent with this and, in combination with the full transmission spectra in Fig. S8-4 demonstrates that DQI results from the interplay between the graphene edge and the molecular connection point.

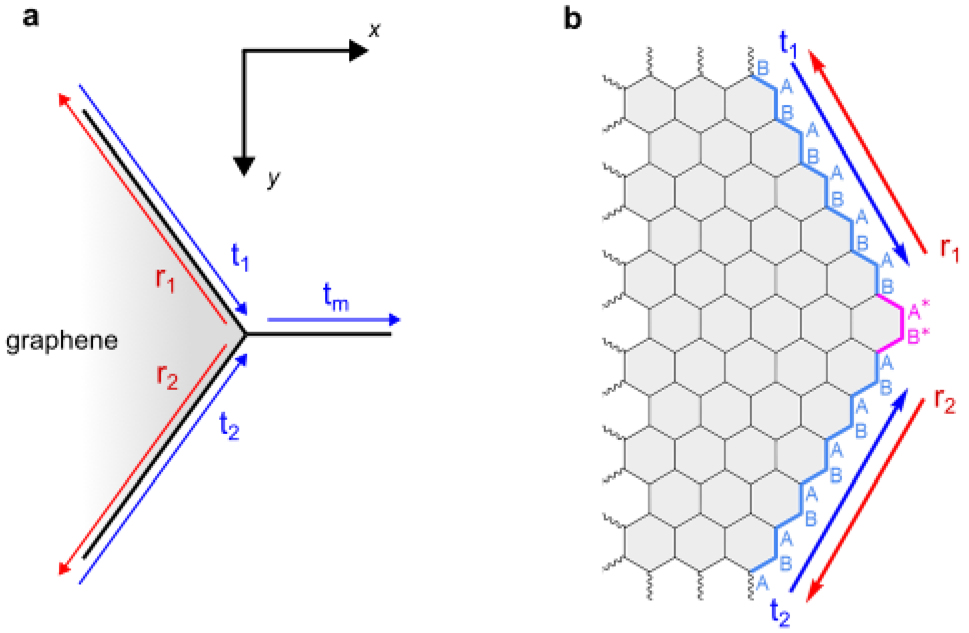

Fig. S8-6 a Model showing the origin of DQI at a graphene/single-molecule interface, where $t_n = C_n \exp i\overrightarrow{k_n}.\vec{r}$ and $r_n = D_n \exp i\overrightarrow{k_n}.\vec{r}$ are transmission and reflection coefficients along the graphene edge and $t_m = C_m \exp(-k_m x)$ is the molecular transmission coefficient. DQI of the graphene edge states at the connecting atom between the graphene and molecule causes the pre-factor, C_m, to go to zero. The edge atoms are labelled by A/B according to their sublattice. b Edge states of a graphene wedge. **b** Edge states of a graphene wedge. Zigzag edges are highlighted in light blue, an armchair edge segment is highlighted in magenta. The blue and red arrows indicate the propagation of wave functions at K and K' valleys, respectively.

As an additional confirmation that the dip in the transmission spectrum results from destructive interference at the molecule-graphene interface, we calculated the atom-to-atom transmission coefficients[15] for the device geometry in Fig. 2 and Fig. S8-4. Fig. S8-7 displays the presence of circular ring currents along the graphene edges and at the connecting atom that reverse direction either side of the anti-resonance, a signature of DQI and its origin at the graphene edge and graphene/molecule interface.[15]

The electroburning procedure to fabricate the graphene nanogap electrodes does not control the edge structure, however we note that test reactions in Fig. S1-3 and described in Supplementary Section 1, show that carboxylic armchair edges are unreactive to amide-coupling chemistry. Whilst the electroburnt graphene nanogap will almost certainly be a mixture of differently shaped protrusions, with the current dominated by tunnelling at the narrowest width,[13] an experimental method to either visualize or preferably control the graphene edge at the atomic level would be required to assign a particular edge geometry to the device.

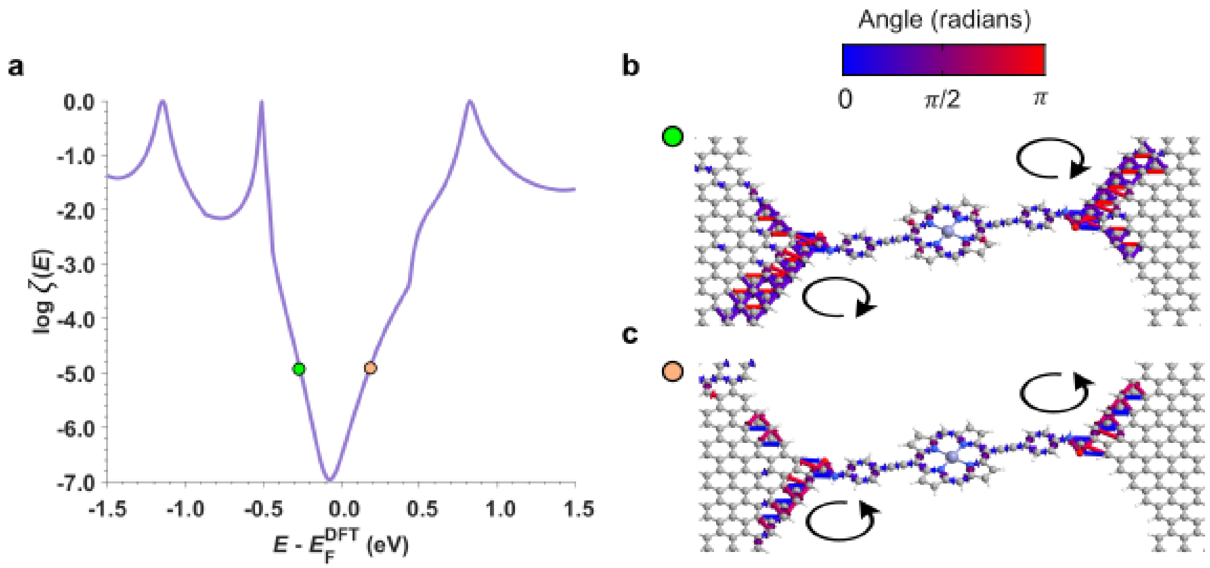

Fig. S8-7 a Transmission spectrum of the single-molecule transistor. **b** and **c** show the transmission pathway through the junction decomposed into atom(i)-to-atom(j) coefficients: $\zeta = \sum \zeta_{ij}$ at V_g = -0.30 V and V_g = 0.25 V respectively. The size of each arrow represents the magnitude of the transmission coefficient, and the colour represents the angle between ζ_{ij} and the transport direction, i.e., those in blue lie along the direction of net transport and those in red oppose it. The presence of cyclic transmission pathways that reverse in direction either side of the transmission dip (indicated by the black arrows) are a signature of DQI originating at the graphene edge/molecule interface.[15]

Supplementary References

1 Grozema, F. C., Houarner-Rassin, C., Prins, P., Siebbeles, L. D. A. & Anderson, H. L. Supramolecular Control of Charge Transport in Molecular Wires. *J. Am. Chem. Soc.* **129**, 13370-13371 (2007). https://doi.org:10.1021/ja0751274

2 Prins, F. *et al.* Room-Temperature Gating of Molecular Junctions Using Few-Layer Graphene Nanogap Electrodes. *Nano Lett.* **11**, 4607-4611 (2011). https://doi.org:10.1021/nl202065x

3 Gehring, P. *et al.* Quantum Interference in Graphene Nanoconstrictions. *Nano Lett.* **16**, 4210-4216 (2016). https://doi.org:10.1021/acs.nanolett.6b01104

4 Limburg, B. *et al.* Anchor Groups for Graphene-Porphyrin Single-Molecule Transistors. *Advanced Functional Materials* **28**, 1803629 (2018). https://doi.org:https://doi.org/10.1002/adfm.201803629

5 Gaussian 16 Rev. C.01 (Wallingford, CT, 2016).

6 Lu, T. & Chen, F. Multiwfn: a multifunctional wavefunction analyzer. *J. Comput. Chem.* **33**, 580-592 (2012). https://doi.org:10.1002/jcc.22885

7 Humphrey, W., Dalke, A. & Schulten, K. VMD: visual molecular dynamics. *J Mol Graph* **14**, 33-38, 27-38 (1996). https://doi.org:10.1016/0263-7855(96)00018-5

8 Yoshizawa, K. An Orbital Rule for Electron Transport in Molecules. *Acc. Chem. Res.* **45**, 1612-1621 (2012). https://doi.org:10.1021/ar300075f

9 Soler, J. M. *et al.* The SIESTA method for ab initio order-N materials simulation. *J. Phys.: Condens. Matter* **14**, 2745 (2002).

10 Perdew, J. P. & Zunger, A. Self-interaction correction to density-functional approximations for many-electron systems. *Phys. Rev. B* **23**, 5048-5079 (1981). https://doi.org:10.1103/PhysRevB.23.5048

11 Ferrer, J. *et al.* GOLLUM: a next-generation simulation tool for electron, thermal and spin transport. *New J. Phys.* **16**, 093029 (2014). https://doi.org:10.1088/1367-2630/16/9/093029

12 Carrascal, D., García-Suárez, V. M. & Ferrer, J. Impact of edge shape on the functionalities of graphene-based single-molecule electronics devices. *Phys. Rev. B* **85**, 195434 (2012). https://doi.org:10.1103/PhysRevB.85.195434

13 García-Suárez, V. M. *et al.* Spin signatures in the electrical response of graphene nanogaps. *Nanoscale* **10**, 18169-18177 (2018). https://doi.org:10.1039/C8NR06123H

14 Valkenier, H. *et al.* Cross-conjugation and quantum interference: a general correlation? *Physical Chemistry Chemical Physics* **16**, 653-662 (2014). https://doi.org:10.1039/C3CP53866D

15 Solomon, G. C., Herrmann, C., Hansen, T., Mujica, V. & Ratner, M. A. Exploring local currents in molecular junctions. *Nature Chemistry* **2**, 223-228 (2010). https://doi.org:10.1038/nchem.546
